# Contactless Assessment of Cerebral Autoregulation by Photoplethysmographic Imaging at Green Illumination

**DOI:** 10.3389/fnins.2019.01235

**Published:** 2019-11-13

**Authors:** Olga A. Lyubashina, Oleg V. Mamontov, Maxim A. Volynsky, Valeriy V. Zaytsev, Alexei A. Kamshilin

**Affiliations:** ^1^Laboratory of Cortico-Visceral Physiology, Pavlov Institute of Physiology, Russian Academy of Sciences, Saint Petersburg, Russia; ^2^Valdman Institute of Pharmacology, Pavlov First Saint Petersburg State Medical University, Saint Petersburg, Russia; ^3^Department of Circulation Physiology, Almazov National Medical Research Centre, Saint Petersburg, Russia; ^4^Faculty of Applied Optics, ITMO University, Saint Petersburg, Russia

**Keywords:** brain microcirculation, vascular tone, cerebral autoregulation, visceral pain, somatic pain, imaging photoplethysmography

## Abstract

Accurate and practical assessment of the brain circulation is needed to adequately estimate the viability of cerebral blood flow regulatory mechanisms in various physiological conditions. The objective of our study was to examine feasibility of the contactless green-light imaging photoplethysmography (PPG) for assessing cerebral autoregulation by revealing the dynamic relationships between cortical microcirculation assessed by PPG and changes in systemic blood pressure caused by visceral and somatic peripheral stimuli. In anesthetized male Wistar rats, the PPG video images of the open parietal cortex (either with unimpaired or dissected dura mater), electrocardiogram, and systemic arterial blood pressure (ABP) in the femoral artery were continuously recorded before, during and after visceral (colorectal distension) or somatic (tail squeezing) stimulation. In the vast majority of experiments with intact and removed dura mater, both spontaneous and peripheral stimulation-evoked changes in ABP negatively correlated with the accompanying alterations in the amplitude of pulsatile PPG component (APC), i.e., an increase of ABP resulted in a decrease of APC and vice versa. The most pronounced ABP and APC alterations were induced by noxious stimuli. Visceral painful stimulation in all cases caused short-term hypotension with simultaneous increase in cortical APC, whereas somatic noxious stimuli in 8 of 21 trials produced hypertensive effect with decreased APC. Animals with pressure 50-70 mmHg possessed higher negative cerebrovascular response rate of ABP-APC gradients than rats with either lower or higher pressure. Severe hypotension reversed the negative ratio to positive one, which was especially evident under visceral pain stimulation. Amplitude of the pulsatile PPG component probably reflects the regulation of vascular tone of cerebral cortex in response to systemic blood pressure fluctuations. When combined with different kinds of peripheral stimuli, the technique is capable for evaluation of normal and elucidation of impaired cerebrovascular system reactivity to particular physiological events, for example pain. The reported contactless PPG monitoring of cortical circulatory dynamics during neurosurgical interventions in combination with recordings of changes in other physiological parameters, such as systemic blood pressure and ECG, has the appealing potential to monitor viability of the cortex vessels and determine the state of patient’s cerebrovascular autoregulation.

## Introduction

Adequate assessment of cerebral hemodynamics is an important task needed to be solved to estimate viability of the cortex and regulatory mechanism. The maintenance of adequate CBF is critical for proper supply of the brain with the necessary oxygen and energy substrates. As known, CBF depends on the blood pressure in cerebral arteries (ABP), the pressure in the cerebral venous system, and resistance of blood flow in small cerebral vessels (Czosnyka et al., 1997; Cipolla, 2009). Blood pressure in cerebral vessels depends on both the cardiac output and resistance of peripheral vessels (Ogoh et al., 2005; Donnelly et al., 2016). Thus, any physiological or pathological event affecting the heart, vasculature, and/or systemic blood pressure as a whole has the potential to alter CBF.

At the level of the brain vessels themselves, CBF is controlled by active changes in vascular tone occurring at the level of the cerebral arterioles, a process referred to as cerebral autoregulation. This is an ability of the brain to maintain relatively constant blood flow despite changes in blood pressure (Paulson et al., 1990; Cipolla, 2009; Fantini et al., 2016). Cerebral autoregulation is a negative feedback loop mechanism that counteracts the mean ABP increase by increasing vascular tone and narrowing the vessels diameter (thus increasing resistance of vessels) that brings CBF to the original level. Conversely, a decrease of ABP results in CBF diminishing whereas the regulatory mechanism decreases the vascular tone leading to vessels dilation to rebalance CBF (Lee et al., 2001; Ozol et al., 2002; Donnelly et al., 2016; Fantini et al., 2016). Physiological origin of cerebral autoregulation is still unclear, with proposed mechanisms invoking myogenic (Ozol et al., 2002; Cipolla, 2009), metabolic (Paulson et al., 1990; Ainslie and Brassard, 2014), and neurogenic processes (Hamel, 2006; Drake and Iadecola, 2007; Donnelly et al., 2016).

Considering importance of CBF regulation, availability of convenient and accurate methods for its assessment in various physiological and pathological conditions is crucial. Various pain conditions are known to cause changes in systemic blood pressure and heart rate implying their influence on cerebral circulation (Sacco et al., 2013; Kyle and McNeil, 2014). It was shown by using different methods (positron emission tomography, functional magnetic resonance imaging, and laser-Doppler flowmetry) that visceral and somatic noxious stimuli are associated with blood flow reactions in widespread brain areas, including cortex (Coghill et al., 1998; Erdos et al., 2003; Dunckley et al., 2005; Derbyshire, 2007). However, the precise impact of different types of pain on local hemodynamics in particular brain regions remains uninvestigated. Recently developed technique that uses an extensive array of optical infra-red sensors (sources and detectors) located at the scalp is capable for measuring the temporary distension of the cerebral arteries caused by the movement of the pulse pressure wave across the vascular system (Fabiani et al., 2014; Tan et al., 2016). It was shown by means of this technique that optically measured pulse amplitude (related to the difference between systolic and diastolic blood pressure) can track both generalized and localized changes in cerebrovascular tone (vasodilation and vasoconstriction) (Tan et al., 2016). The technique with array of optical sensors allows quantitative characterization of the CBF dynamics in real time. However, this method is hardly applicable to monitor cerebral hemodynamics during brain surgery. At the same time, assessing the dynamic parameters of CBF is an important task whose solution could characterize quality of surgical interventions on the open brain and determine the patient’s prognosis.

A wide variety of optical methods is used to evaluate non-invasively CBF in real time. Among them, the methods based on video recordings of the exposed brain under different illumination are very popular due to their low cost, simplicity of implementation, and no use of any dies (Bouchard et al., 2009; Ma et al., 2016). These methods are referenced to as OISI revealing slowly varying changes in the recorded video images of the open brain, which are assumed to be proportional to changes in blood volume (Frostig et al., 1990; Malonek and Grinvald, 1996). Temporal modulation of OISI signals at the heartbeat frequency is typically considered as an artifact which have to be filtered out to increase the SNR (Stewart et al., 2013). In contrast, heartbeat related modulation is the primary source of information in imaging PPG systems (Allen, 2007; Kamshilin et al., 2011). These systems are widely used for assessment of cardiovascular parameters from video images of the human skin but not enough attention was paid to use them for studying brain hemodynamics.

Here, we report experimental data that demonstrate feasibility to assess cerebral hemodynamic after processing video frames of the open brain that were recorded by the custom-made imaging PPG system at incoherent green illumination. The aim of our research was to reveal a relationship between variations of the systemic blood pressure (caused particularly by different pain stimuli in rats) and changes in CBF assessed by contactless, non-invasive, and cost efficient optical method.

## Materials and Methods

### Animals and Ethics

Experiments were performed with the open cortex of a rat’s brain. Adult (8–14 weeks) male Wistar rats (*n* = 11), 327 ± 53 g were used in this study. The animals were housed 2–5 per cage under standard laboratory conditions (12:12 light-dark cycle) with free access to food and water. They were deprived of food, but not water, for 16 h before each experiment. The Institutional Animal Care and Use Committee of the Pavlov Institute of Physiology of the Russian Academy of Sciences approved all experimental procedures. The experiments were conducted in accordance with the guidelines set by the European Community Council Directives 86/609/EEC, with the Ethical Guidelines of the International Association for the Study of Pain, and EU Directive 2010/63/EU for animal experiments. After the end of each experiment, the rat was sacrificed by intravenous injection of a lethal dose of urethane (>3 g/kg). All possible efforts were made to minimize animal suffering and to use only the number of animals necessary to produce reliable data.

### Animals Preparation

Surgical procedures were performed under deep anesthesia with a mixture of urethane (800 g/kg, i.p.; ICN Biomedicals, Aurora, OH, United States) and α-chloralose (60 mg/kg; MP Biomedicals, Solon, OH, United States) as it was described in detail previously (Lyubashina et al., 2016). Briefly, the left femoral artery and left femoral vein were cannulated for continuous monitoring of blood pressure and drug administration, respectively. The trachea was exposed and a tracheal cannula was inserted for measurements of respiratory airflow and end-tidal carbon dioxide. Dorsal parts of the left frontal and parietal skull bones were removed so that maximum care was taken to preserve integrity of meninges and prevent bleeding. The measurements were performed in paired experiments: first, the response of cerebral cortex vessels was evaluated using video recordings through dura mater covered with warm mineral oil, then the dura mater was removed and the entire measurement cycle was repeated. In this case warm saline was dripped over the exposed area. Since the absence of dura mater is typical of brain neurosurgical operations, these paired experiments were planned to determine the possibility of contactless measuring blood pulsations of the cerebral cortex in various situations. The anesthetized rat was mounted into a stereotactic frame for craniectomy and remained there during video recording after the surgery. Body temperature was maintained at approximately 38°C with a heating pad.

### Types of Stimulations

Two kinds of stimulations (visceral and somatic) were used in our experiments. To induce visceral nociception, a colorectal distension was performed as described previously (Lyubashina et al., 2018). It was produced by inflating a 7 cm long, 2 cm diameter flexible latex balloon, which was inserted transanally and kept in position (the end of the balloon 1 cm proximal to the anus) by taping the connecting catheter to the tail. While for innoxious (physiological) visceral stimulation the balloon was inflated to a pressure of 40 mmHg, for visceral pain induction, the colorectal balloon was rapidly inflated to a pressure of 90 mmHg. Innocuous (tactile) somatic stimulation was delivered to the rat’s tail with forceps. Somatic nociception was caused by firmly squeezing the base of the tail by surgical forceps with a fixed lock mechanism as it was described previously (Lyubashina et al., 2019).

### Experimental Protocol

After the surgery, the rat was kept in rest during 40 min to minimize the effect of postsurgical reaction. The total duration of the experiment was 30 min. It consisted of four successive series in which the following stimulations were applied: (1) innoxious visceral, (2) tactile somatic, (3) painful visceral, (4) painful somatic. The minimum time interval between series was 5 min. In each series, the data containing the cortex images, ECG, and ABP were recorded continuously while the rat was consistently in the following physiological states: baseline (90 s), stimulation for 1 min, and relaxation (90 s).

### Experimental Arrangement

**FIGURE 1:**
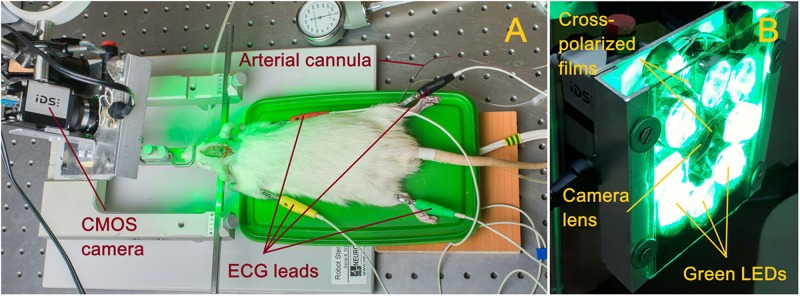
Photographs of the experimental setup **(A)** and imaging PPG system **(B)**.

Parameters of cortical blood flow were measured by using home-made imaging PPG system (Kamshilin et al., 2016). Photographs of the experimental setup and PPG system are shown in Figure 1. Open brain of anesthetized rat was illuminated by the green light generated by LEDs operating at the wavelength of 530 ± 25 nm as shown in Figure 1A. This illumination was chosen because several research groups working with the reflection-mode PPG reported that the largest intensity modulation at the heartbeat frequency was observed at the green light modulation (Verkruysse et al., 2008; Kamshilin et al., 2011; Fallow et al., 2013). PPG-system design was described in details in our recent paper (Kamshilin et al., 2018). Briefly, it consists of a digital monochrome CMOS camera (10-bit model GigE uEye UI-5220SE of the Imaging Development Systems GmbH) integrated with an illuminator with a polarization filter. A photograph of the system is shown in Figure 1B. All LEDs were assembled around the camera lens (25 mm focal length) providing uniform illumination of the open brain area. Incoherent light generated by LEDs was linearly polarized by means of a film polarizer (Edmunds Optics, 0.18 mm thickness) attached to the LED assembling. Another film polarizer was attached to the camera lens so that transmission axis was oriented orthogonal to the polarization vector of the illuminating light. Extinction ratio of the used polarizing film was 9000:1. As known, polarization filtration reduces specular reflections and motion artifact influence on the detected signals (Sidorov et al., 2016).

All videos were recorded at 100 frames per second with resolution of 752 × 480 pixels and saved frame-by-frame in the hard disk of a personal computer. Such a frame rate is sufficient to solve the research task since it provides eight or more readings per cardiac cycle of a rat. ECG and ABP in the femoral artery were recorded simultaneously with video. Digital electrocardiograph (model KAP-01-“Kardiotekhnika-EKG,” Incart Ltd., Saint Petersburg, Russia) operating at the sample frequency of 1 kHz was used to record ECG synchronized with video frames with accuracy of 1 ms. ABP in the femoral artery was continuously monitored with a pressure transducer (MLT844, AD Instruments Inc., Colorado Springs, United States) at the sample frequency of 10 kHz and recorded in the personal computer using Spike2 software (Cambridge Electronic Design, Cambridge, United Kingdom).

### Data Processing

All recorded video frames, ECG, and ABP were processed off-line by using custom software implemented in the Matlab^®^ platform. To evaluate CBF dynamics we used an amplitude of the pulsatile component (APC) of the photoplethysmographic waveform that shows modulation of the light reemitted from the brain at the heartbeat frequency (Allen, 2007). Spatial distribution of APC over the cortex was calculated using an algorithm described in details in our recent paper (Kamshilin et al., 2018). Briefly, the algorithm includes the following steps.

**FIGURE 2:**
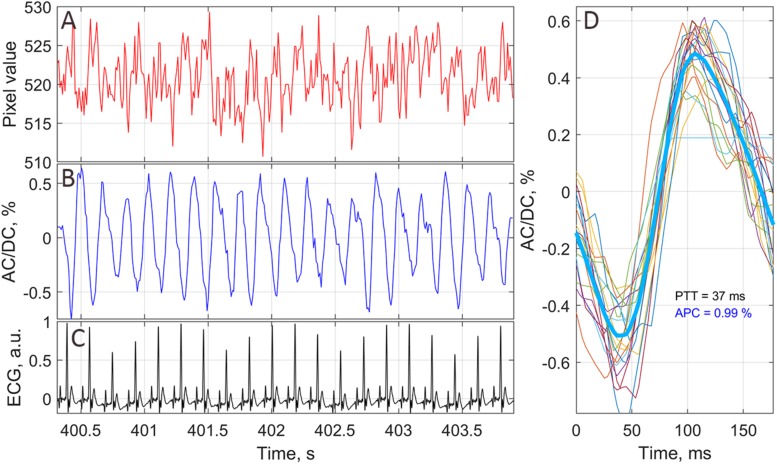
An example of PPG waveform from one of the small chosen ROIs. **(A)** Raw signal averaged in the ROI of 3 × 3 pixels without any stabilization or filtration. **(B)** PPG waveform as AC/DC ratio after image stabilization procedure and band-pass filtering. **(C)** ECG signal synchronously recorded with video frames. **(D)** One-cardiac-cycle waveform (thick blue line) as averaging of 20 successive cardiac cycles (thin colored lines). The waveform of each cardiac cycle plotted so that it starts at corresponding R-peak of ECG.

**FIGURE 3:**
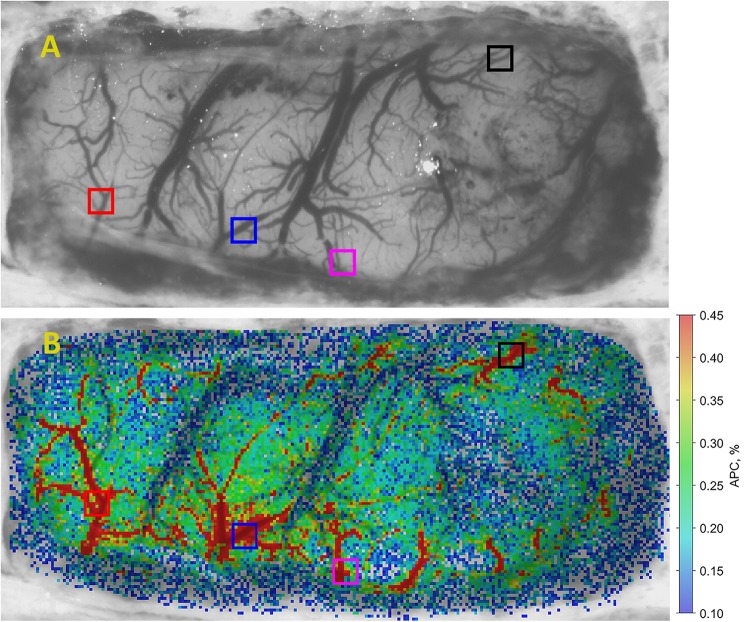
An example of APC mapping in brain cortex. **(A)** One of the recorded video frames of the open rat’s brain, and **(B)** spatial distribution of APC overlaid with initial image. The color scale on the right shows APC in percent. Colored squares show location of selected big ROIs in which APC dynamics has been estimated.

• First, the images were digitally stabilized to compensate involuntary motions of rat’s brain using previously described algorithm (Kamshilin et al., 2018). Considering that different parts of the brain image are displaced stochastically and heterogeneously, we divided the whole image on the segments of 64 × 30 pixels, and compensated the motion of each segment independently. We assumed that the signal variations have two components: PPG and the motion-related parts. The motion-related component is proportional to the image gradient and lateral offset. The lateral offset was estimated in every segment by optical flow algorithm (Kearney et al., 1987) using gradient method and then the motion-related signal component was reconstructed and subtracted from the original signal.
• Second, an area of the open brain was covered by small regions of interest (ROI) sizing 3 × 3 pixels, which corresponds to the area of 40 × 40 μm^2^ at the rat’s cortex. Each ROI was chosen to have a common border with adjacent ROIs without overlapping.
• Third, we calculated PPG waveform as frame-by-frame evolution of average pixel value in every small ROI. An example of an unfiltered waveform averaged within one of the ROIs over raw, non-stabilized frames is shown in Figure 2A. For clarity of presentation, only 20 cardiac cycles are shown in this graph. As seen, the waveform consists of AC caused by pulsatile character of blood flow, and DC. Both components are proportional to the incident light intensity. By calculating AC/DC ratio for the pixels averaged within small ROIs over the stabilized frames, we compensate unevenness of the incident illumination taking into account that both components are proportional to the light intensity for green-light illumination (Liu et al., 2014). After subtracting the unity from the calculated ratio and inverting its sign, we obtained a waveform that correlates positively with that of ABP (Allen, 2007; Kamshilin et al., 2018). All PPG waveforms were filtered to remove noise and DCs by means of a band-pass filter (0.12–200 Hz), which was implemented using the *filtfilt* function in Matlab^®^ to perform zero-phase digital filtering of the waveforms. Filtered PPG waveform is shown in Figure 2B whereas the waveform of synchronously recorded ECG is shown in Figure 2C. One can see that each PPG pulse follows the respective R-peak of ECG signal.
• Fourth, in every small ROI, we assessed a mean PPG pulse from which the parameter APC was estimated. Algorithm of the mean PPG pulse estimation is illustrated in Figure 2D. In this graph, we plotted together (by thin colored lines) PPG pulses of 20 subsequent cardiac cycles so that each respective R-peak of the ECG is at the beginning of the time scale (Kamshilin et al., 2019). Averaging of these 20 pulses results in the mean PPG pulse shown in Figure 2D by thick blue line. Parameter APC was calculated as the difference between the maximum and minimum of the mean pulse after each series of 20 cycles. The number of cardiac cycles (20) used for averaging allowed us to reduce the effect of respiration, which influenced the amplitude of particular PPG pulses. Typically, 20 cardiac cycles contains more than two respiratory cycles. The time interval of 20 subsequent cardiac cycles defines the temporal resolution with which we assessed variations of APC. It depends on the rat’s heart rate and varies for our rats from 1.6 to 3.5 s. To assess APC dynamics, we calculate this parameter once each 20 cardiac cycles. Since it was calculated in every small ROI, we were able to mapping this parameter on the image of the rat’s cortex for visualization of its spatial temporal variations caused by the functional stimulation. We defined here the SNR of the PPG waveform as the ratio of the mean amplitude during 20 cardiac cycles to the STD of the amplitude for particular PPG pulses.
• Fifth, to quantify the APC dynamics, we selected four big ROIs nearby different cerebral arteries (see an example of such selection in Figure 3A). It was found that the pulsatile component of PPG waveform usually has higher amplitude in these areas (Kamshilin et al., 2019). Each big ROI contains 9 × 9 small ROIs or 27 × 27 pixels that corresponds to the region of 0.36 × 0.36 mm at the rat’s cortex. With each rat, we performed four different functional stimulations. The whole experiment lasted half an hour. During the experiment, the image of the rat brain was sometimes shifted. This shift was compensated after each 20 cardiac cycles so that the net of small ROIs remained in the same relative position in respect to the blood vessels. Therefore, the position of big ROIs relative to the cortical vessels remained unchanged both during each functional test and in all four tests.

### Statistical Analysis

In total, 21 experiments were carried out with 11 rats: reaction on four different physiological stimuli was monitored in all of them while keeping the dura mater intact, whereas 10 rats were measured after the dura mater was removed. In every experiment, we assessed the dynamics of APC in four big ROIs. Therefore, 84 APC traces were analyzed. In each experiment, four courses of APC in big ROIs were compared with a single course of ABP. To assess the dynamics of ABP during physiological stimuli, we performed a comparative analysis using the dependent *t*-test for paired samples (parametric method) evaluated during 13 s before and 13 s just after the stimuli. Pearson’s parametric correlation was used to assess the relationship of ABP and APC. While calculating the Pearson coefficient of correlation, we compared the ABP trace with each of the APC traces, all of them recorded during 3 min: 60 s before, 60 s during, and 60 s after the physiological stimulation. To identify significant fluctuations in ABP, we used the rule of three STD calculated for the baseline before physiological stimuli.

## Results

### Spatial Distribution of the Pulsatile Component

An example of spatial distribution of the parameter APC over the open rat’s cortex is shown in Figure 3B. The APC map in Figure 3 is coded in pseudo colors with the scale shown on the right. As seen, pulsating arteries are clearly distinguished from the veins because of much higher amplitude of the pulsatile component synchronized with cardiac activity. Big ROIs in which the APC dynamics was monitored were selected on the location of arteries since these areas are characterized with larger amplitude of pulsations. These ROIs are shown by colored squares in Figure 3B. Similar maps were calculated with the step of 1.6–3.5 s for each series of functional stimulation. Note that systemic blood pressure was monitored simultaneously with video recordings from which spatial distribution of cortical blood pulsations was evaluated.

### Negative Correlation Between APC and Systemic Blood Pressure

**FIGURE 4:**
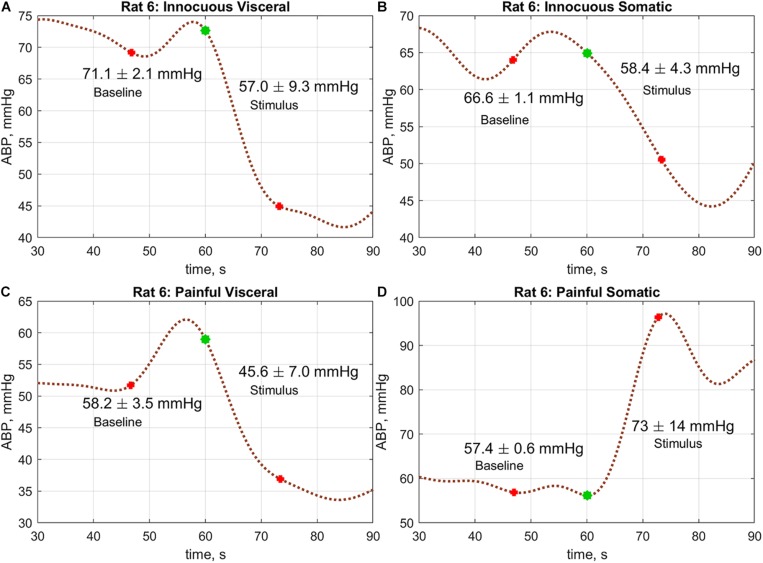
An example of the ABP responses on different physiological stimuli measured in rat#6: **(A**) innocuous visceral stimulation, **(B)** tactile somatic, **(C)** painful visceral, and **(D)** painful somatic. Blue marks in the graphs show the moments when the stimulation begins. Red marks show the windows borders within which the change of the mean ABP was estimated.

To assess the reaction of the ABP on a physiological stimulus, we selected 13-s trace recorded prior the stimulus application, and compared the ABP averaged over this trace with that averaged over the next 13 s just after stimulus application. The choice of the averaging windows with duration of 13 s was due to ideas about the autoregulation of hemodynamics, which can smooth out the effect of the stimulus at later stages. The hemodynamic responses on four different stimuli for one of the rat are shown in Figure 4.

A comparative analysis revealed that the hemodynamic response in rats was significantly different in visceral and somatic stimuli. Moreover, the reaction did not depend on the intensity of stimulation. It is worth noting that in the group average, no difference in responses to tactile and painful stimuli was revealed in the case of somatic stimulation: 67.0 ± 16.8 vs. 65.0 ± 17.2 mmHg; *P* = 0.20 and 63.7 ± 17.9 vs. 63.6 ± 16.1 mmHg; *P* = 0.97, respectively. However, significant diminishing of the mean ABP was observed in the case of innocuous visceral stimulus: 67.5 ± 17.1 mmHg before stimulus vs. 61.5 ± 16.8 mmHg after, *P* < 0.001. Such a drop of the mean ABP was even more pronounced in the case of painful visceral stimulation: 64.2 ± 17.9 vs. 55.2 ± 16.7 mmHg, *P* < 0.001. At the same time, there were no differences in ABP responses on stimuli depending on the presence of a dura mater.

Nevertheless, the observed absence of the ABP reaction on the painful somatic stimulation in the group average might be explained by qualitative difference in rat’s reaction on this stimulus. As seen in Figure 5 and Supplementary Figures S1–S21, some rats respond to painful somatic stimulation by a decrease in ABP, whereas others – by its increase. While visceral stimulation of all rats results in a statistically significant decrease in systemic arterial pressure (Figures 5A,C,E), somatic stimulation leads to an increase of the mean blood pressure in 8 out of 21 cases (Figures 5B,F and Supplementary Figures S4, S5, S8–S13).

**FIGURE 5:**
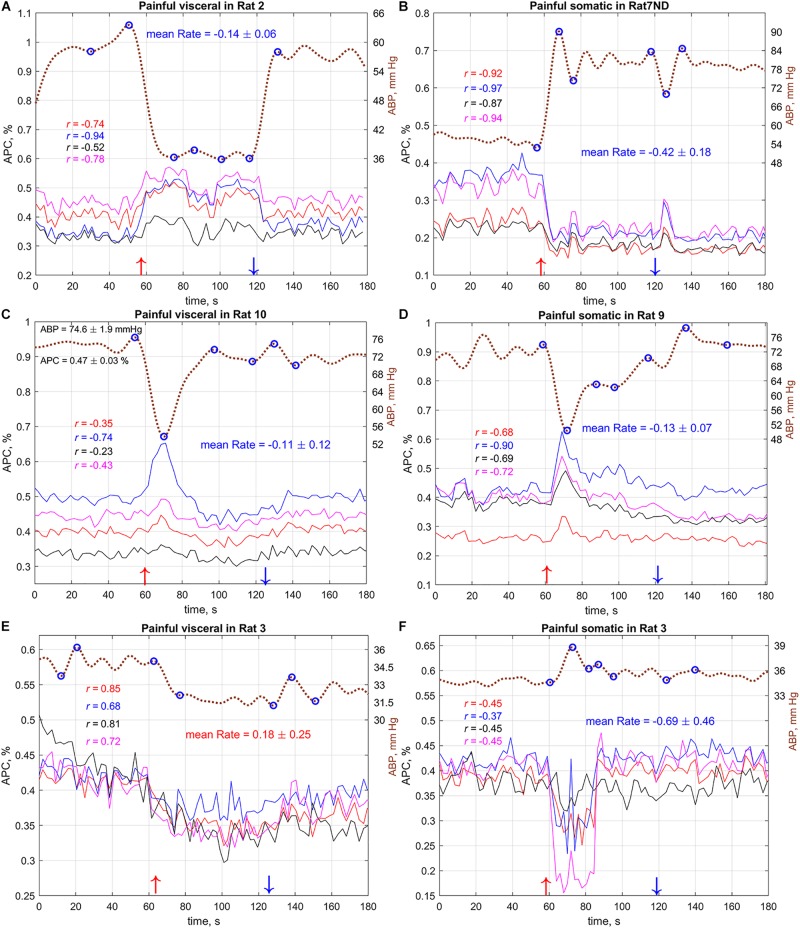
Typical **(A–C)** and atypical **(D–F)** dynamics of the systemic blood pressure and amplitude of pulsatile component in cerebral arteries of several rats during visceral and somatic stimulation. Dashed brown curves show dynamics of the mean arterial blood pressure with the labels on the right. Solid colored lines show APC measured in big ROIs selected in different areas of the cortex. Marker “ND” in the rat’s name above the graphs means that the measurements were carried out without dura mater. Color of a correlation coefficient corresponds to the color of APC curve in each panel. Red and blue arrows nearby the *X*-axes show moments of the beginning and end of stimulation, respectively. The complete set of the responses for all 11 rats (with and without dura mater) is available in Supplementary Figures S1–S21.

As one can see in Figure 5, variations of systemic blood pressure in the most cases are opposite to the changes in pulsation amplitude of cerebral arteries. This was confirmed by the correlation analysis between ABP and APC traces. The negative sign of correlation observed in some cases means inverse relationship of these parameters. For example, in the case demonstrated in Figure 5B, the correlation between APC averaged in the selected ROIs and systemic pressure is very strong: −0.97 < *r* < −0.87, *P* < 0.001. However, APC positively correlates with mean arterial pressure in few exceptions; one of them is shown in Figure 5E for the case of painful visceral stimulation of rat#3. Note that the mean pressure of this rat (Figure 5E) was significantly lower than that of others. Interestingly, the painful somatic stimulation applied 7 min later to the same rat and accompanied by an increase in blood pressure had revealed the negative correlation between systemic pressure and APC (Figure 5F). This allowed us to suggest that, despite critical hypotension, feedback on the separate increases in blood pressure is maintained.

**FIGURE 6:**
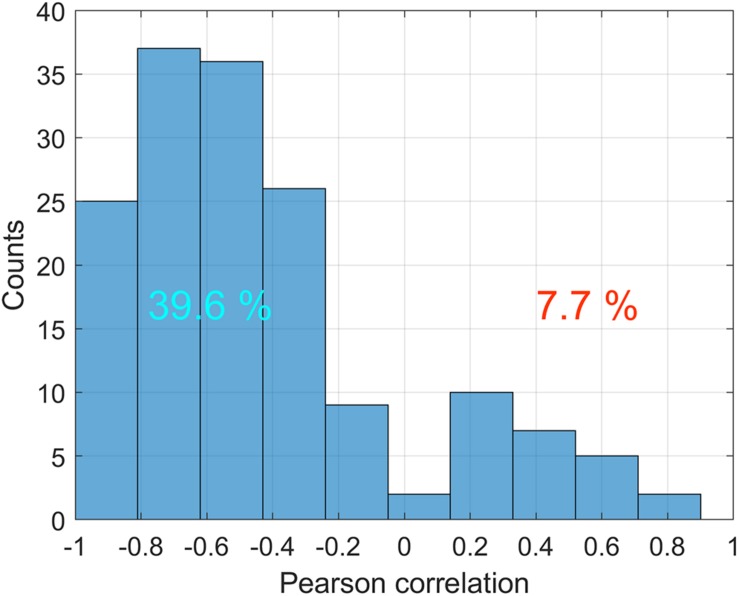
Distribution of correlation coefficients between ABP and APC in various big ROIs during different stimulation sessions for all 11 rats. Only statistically significant cases are counted. Numbers on the positive and negative parts show percentages of statistically significant correlations with positive and negative signs, respectively.

Correlation analysis of the relationship between ABP and APC was carried out for PPG waveforms calculated in each big ROI in every stimulation sessions for all rats. The total number of traces for the correlation analysis was *n* = 336. The histogram of the correlation coefficients between these variables is shown in Figure 6. It is seen that statistically significant negative correlation between the dynamics of ABP and APC was observed in 39.6% of total cases, whereas the positive correlation between these parameters was observed in 7.7% of the cases, i.e., 5 times less. These data indicate that in most, but not all cases, the amplitude of the pulsatile component is a parameter characterizing the feedback mechanism that corrects CBF in response to fluctuations in systemic blood pressure.

It should be noted that four big ROIs were selected in vicinity of different cerebral arteries for each rat. The correlation analysis has revealed in the most of cases (15 of 21 experiments) substantial positive pairwise correlation between APC dynamics assessed nearby different arteries. For example, the Pearson coefficient varied from 0.65 to 0.95 (*P* < 0.05) in the session of painful somatic stimulation for rat #8 with dura mater. Much greater variations in the correlation coefficient were observed in the same session for the same rat but without dura mater: between 0.23 and 0.81. However, resection of dura mater in other rats led to the opposite effect: 0.23 < *r* < 0.85 with dura mater vs. 0.92 < *r* < 0.98 without dura mater in rat #7. We believe that this behavior shows that, with the general tendency for the responses of cerebral arteries on painful stimuli to be similar, some of them have their own characteristics. Nevertheless, no correlation was observed (*P* > 0.05) between some pairs of the ROIs in 6 of 21 experiments possibly due to the impaired mechanism of autoregulation.

### Cerebrovascular Response Rate

To evaluate the response of APC parameter on systemic pressure variations quantitatively, we found the pressure jumps exceeding 7% of the average systemic pressure in each session of the physiological stimulation. The choice of such a threshold was determined by two factors: it had to exceed small spontaneous changes of APC and reflect pronounced variations, including those induced by physiological stimuli. To separate spontaneous changes, we used the rule of three STD. Since STD for the group average was 2.3% of the mean value, we adopted the threshold of 7%. Note that particular fluctuations of ABP either at rest or during stimuli were much higher. The time borders of such jumps are marked by blue circles in every curve of the systemic pressure dynamics (Figure 6). Within these borders, we calculated the ratio of the mean gradient of APC (normalized to the mean value of APC) and the gradient of the blood pressure, which was adopted here as a CRR measured in percent per mmHg. Negative sign of CRR means that an increase of the systemic pressure results in decrease of APC and vice versa.

**FIGURE 7:**
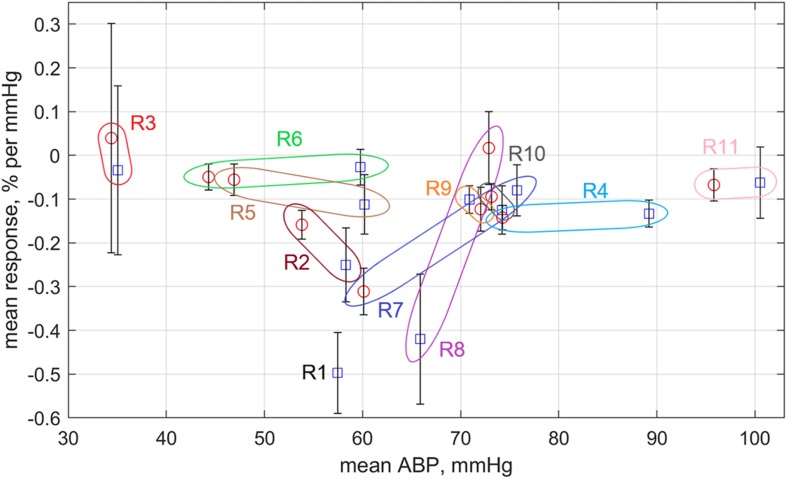
Response rate of mean APC measured in the cortex on variations of the mean systemic blood pressure. Blue squares are data obtained for rats before dura mater resection, whereas red circles are for the rats after the resection. All data are sown as the mean value ± standard error of the mean. Colored loops with the rat’s marker bind the square and circle measured for the same rat.

Four sequential sessions of physiological stimulations were carried out for each rat. First series of stimulations was applied to the open rat’s brain with dura mater and another series – to the same rat but after dura mater resection. In the most cases of innocuous visceral and tactile somatic stimulations, significant pressure changes (>7%) occurred as unrelated with the external impact. The parameter CRR was estimated for these jumps, as well. For each series of four stimulations we calculated the mean response rate and plotted it as a function of the averaged systemic pressure in Figure 7. Blue squares in this plot correspond to the rat with dura mater, and red circles – without. Colored loops bind each pair of points measured for the same rat before and after dura mater resection.

One can see that the response rate is negative for the most of the experimental series (Figure 7). Exceptions are the rat #3 having very low systemic pressure and rat #8, which arterial pressure exceptionally grew up after dura mater resection (for the most of rats this resection resulted in diminishing of the systemic pressure). Rats with systemic pressure between 50 and 70 mmHg possess much high negative CRR than the rats with either lower or higher pressure. It is worth noting that CRR in rat #7 with relatively high pressure before dura mater resection has significant increase of CRR in absolute value after resection due to diminishing of the systemic pressure. In contrast, CRR in rat #8 (with moderate systemic pressure) before resection was one of the highest negative but became positive after resection probably because of the pressure increase. SNR of PPG waveforms was the lowest in the Rat #6 that probably explains its lower negative CRR in spite of the moderate systemic pressure.

## Discussion

Cerebral autoregulation is usually assessed by direct measurements of CBF and mean ABP with subsequent analysis of their temporal courses to reveal their relationship (Myburgh, 2004; Panerai, 2008; Fantini et al., 2016). Various methods are applied for measurement and quantification of this relationship. Most of the current studies on cerebral autoregulation are carried out using transcranial Doppler ultrasound technique that provides non-invasive continuous CBF monitoring by measuring velocity of red blood cells in large brain arteries (Myburgh, 2004; Fantini et al., 2016). The list of other techniques is following. Positron emission tomography (PET) that assesses dynamics of radioactive diffusible contrast agent delivery as a measure of CBF (Fantini et al., 2016). Single photon emission computed tomography, which yields brain perfusion indices reflecting CBF (Asenbaum et al., 1988). Magnetic resonance imaging, the source of signals in which is either the magnetic field gradient between the capillaries containing contrast agent (Lia et al., 2000) or the surrounding tissue or magnetically labeling water in major arteries in the brain (Golay et al., 2004). Diffuse correlation spectroscopy that measures temporal fluctuations of near-infrared laser light scattered by moving red blood cells, which relative changes relate to CBF (Buckley et al., 2014). Functional near infrared spectroscopy that is a diffuse optical method that yields the balance between brain tissue concentrations of oxyhemoglobin and deoxyhemoglobin providing a measure of intravascular oxygenation linked to CBF (Scholkmann et al., 2014). However, all above listed methods of CBF evaluation require sophisticated equipment that is rather expensive. In contrast, the proposed technique is characterized by low cost of its component and ease of use providing good prospects for its application in biomedical research and clinical practice.

Our experiments have shown that in the vast majority of cases, both the spontaneous jumps of the systemic blood pressure and its changes in response to either visceral or somatic stimulation in anesthetized rats lead to counter-phase variations of the APC of the PPG waveform measured in the rat’s cortex. These observations can be explained by a reflex change in the tone of cerebral vessels in response to changes in perfusion pressure. Such a behavior of the vascular tone in general corresponds to the behavior of the cerebrovascular reflex, the main physiological meaning of which is aimed at maintaining the constancy of cerebral perfusion under conditions of fluctuating systemic blood pressure. In other words, APC follows the local vascular resistance that is varying under the influence of systemic arterial pressure. Therefore, the APC of PPG waveform can be considered as a measure of the cerebral vascular tone.

At the same time, disappearance of the negative feedback between perfusion and ABP in rat #3 is most likely due to the disruption of autoregulation under conditions of pronounced reduction in blood pressure. Such a behavior corresponds to a clinical situation caused by stable systemic hypotension when the cerebral vessels are maximally dilated against the background of a pronounced decrease in blood pressure so that further fluctuation of blood pressure does not lead to a significant additional decrease in vascular tone. In turn, the negative feedback was lower also in animals with systemic ABP higher than 70 mmHg, suggesting the hypertension-induced impairment of cerebrovascular reflex. The most efficient negative feedback observed in the range of 50-70 mmHg of systemic ABP seems to be quite logical in view of previously published results (Springborg et al., 2002). These results, taken together, indicate the usefulness of the PPG method for identifying pathological alterations in the mechanism of cerebrovascular regulation under various conditions including dramatic changes (both a fall and an increase) of systemic blood pressure.

Our study has disclosed for the first time that visceral and somatic types of pain lead to preferentially opposite changes in the parietal cortex regional CBF. Previously the unidirectional circulatory responses of this area to pain signals from internal and external parts of the body were proposed (Strigo et al., 2003; Apkarian et al., 2005). In contrast, the use of more sensitive PPG imaging technique has allowed us to reveal the difference. Currently available in the literature brain imaging data on the features of visceral and somatic pain perception were obtained using different techniques and methodologically diverse approaches to functional stimulations. Therefore, slightly different results were reported on the characteristics of the activation of certain regions of the cortex in response to visceral and somatic pain. Findings of this work compliment and amplify the available data on differential perception of visceral and somatic pain in the brain (Strigo et al., 2003; Apkarian et al., 2005; Dunckley et al., 2005; Derbyshire, 2007; Johns and Tracey, 2009; Gebhart and Bielefeldt, 2016).

The observed opposite effect of visceral and somatic noxious inputs on cortical vasculature can be mediated by several potential mechanisms. For instance, previous studies, including our, have revealed that visceral and somatic pain stimuli produce different patterns of neuronal activity in the caudal ventrolateral medulla (Lyubashina et al., 2019), nucleus of the solitary tract, locus coeruleus (Han et al., 2003), and raphe nucleus (Brink and Mason, 2003). On the one hand, prevailing difference between visceral and somatic pain-induced alterations in systemic ABP (and in concomitant opposite changes in CBF) can be caused by diverse activation of these brainstem areas. It was shown that these areas mediate cardiovascular responses to nociceptive events (Elam et al., 1986; Boscan and Paton, 2001; Lima et al., 2002) as being intimately involved in descending control of the spinal sympathetic preganglionic neurons as well as in modulation of the medullary vagal motor neurons (Benarroch, 2001). On the other hand, neurons of the abovementioned brain regions are known to project to cortical microvessels to directly control local blood flow by release of various vasoconstrictor and vasodilator neurotransmitters (Hamel, 2006; Drake and Iadecola, 2007; Cipolla, 2009). Therefore, the modality specific excitation of such structures by visceral painful stimuli can result in the release of vasoactive substances (e.g., catecholamines) (Han et al., 2003; Wang et al., 2009) with the associated changes in cerebral vascular tone and, as a consequence, in cortical blood flow, which may differ from those occurring during somatic pain (Erdos et al., 2003).

In consensus with conclusions of recent studies devoted to CBF imaging by fiber array of optical infra-red sensors (Fabiani et al., 2014; Tan et al., 2016), we assume that increase in the amplitude of cortical pulsatile PPG component relates to distension of the cerebral vessels. Consequently, it allows the blood to flow more easily into the tissue to compensate CBF diminished due to the painful stumuli-induce hypotension. In turn, the reported decrease in the pulsatile amplitude presumably represents a vasoconstriction that results in greater resistance for the blood flowing out of the larger upstream arteries to counteract an increase in CBF due to the stimulation-evoked hypertension.

In summary, we have developed a novel method for contactless, dynamic assessment of spatial temporal variations of blood pulsations in cerebral vessel related to changes in blood supply to the cortex caused by physiological stimuli. It was done by video recording of an open brain illuminated by incoherent green light simultaneously with ECG and ABP. We hypothesize that the parameter APC is a measure of cerebral vascular tone that varies in response to fluctuations in systemic blood pressure. In the case when the autoregulation is disrupted by systemic hypotension, the correlation between ABP and APC reverts the sign. The reported contactless PPG monitoring of cortical circulatory dynamics during neurosurgical interventions in combination with synchronized recordings of systemic blood pressure and ECG, has the appealing potential to determine the state of patient’s cerebrovascular autoregulation. Moreover, responses of cerebral hemodynamics to different pain-producing manipulations can be revealed, helping thus to prognosticate individual outcomes and optimize treatment to a given patient’s need. The proposed method may also play a diagnostic role in extra-cranial cardiovascular disorders and chronic pain conditions, elucidating the pathology-induced alterations in cerebral regulatory mechanisms.

## Data Availability Statement

All datasets generated for this study are included in the article/Supplementary  Material.

## Ethics Statement

The animal study was reviewed and approved by the Institutional Animal Care and Use Committee of the Pavlov Institute of Physiology, Russian Academy of Sciences.

## Author Contributions

OL conceived and designed the study, provided preparation of animals, collected the experimental data, and wrote, reviewed, and edited the manuscript. OM drafted the manuscript, revised the data analysis, and wrote and reviewed the manuscript. MV designed and manufactured the optical – electronic part of the experimental setup, and collected the experimental data. VZ designed the software for images acquisition and collected the experimental data. AK conceived and designed the study, administered the project, performed the data analysis, and wrote, reviewed, and edited the manuscript. All authors provided critical feedback and helped to shape the research, analysis, and manuscript.

## Conflict of Interest

The authors declare that the research was conducted in the absence of any commercial or financial relationships that could be construed as a potential conflict of interest.

## Abbreviations

ABP: arterial blood pressure
AC: alternating component
APC: amplitude of pulsatile component
CBF: cerebral blood flow
CMOS: complementary metal-oxide-semiconductor
CRR: cerebrovascular response rate
DC: slowly varying component
ECG: electrocardiogram
LED: light-emitting diode
OISI: optical intrinsic signals imaging
PPG: photoplethysmography
ROI: region of interest
SNR: signal-to-noise ratio
STD: standard deviation.
