# Supplementary material for: Contactless Assessment of Cerebral Autoregulation by Photoplethysmographic Imaging at Green Illumination
Source: Front Neurosci. 2019 Nov 13;13:1235. doi: 10.3389/fnins.2019.01235 (PMC6863769; doi:10.3389/fnins.2019.01235)
Supplement: Supplementary file 1 [file Data_Sheet_1.PDF]

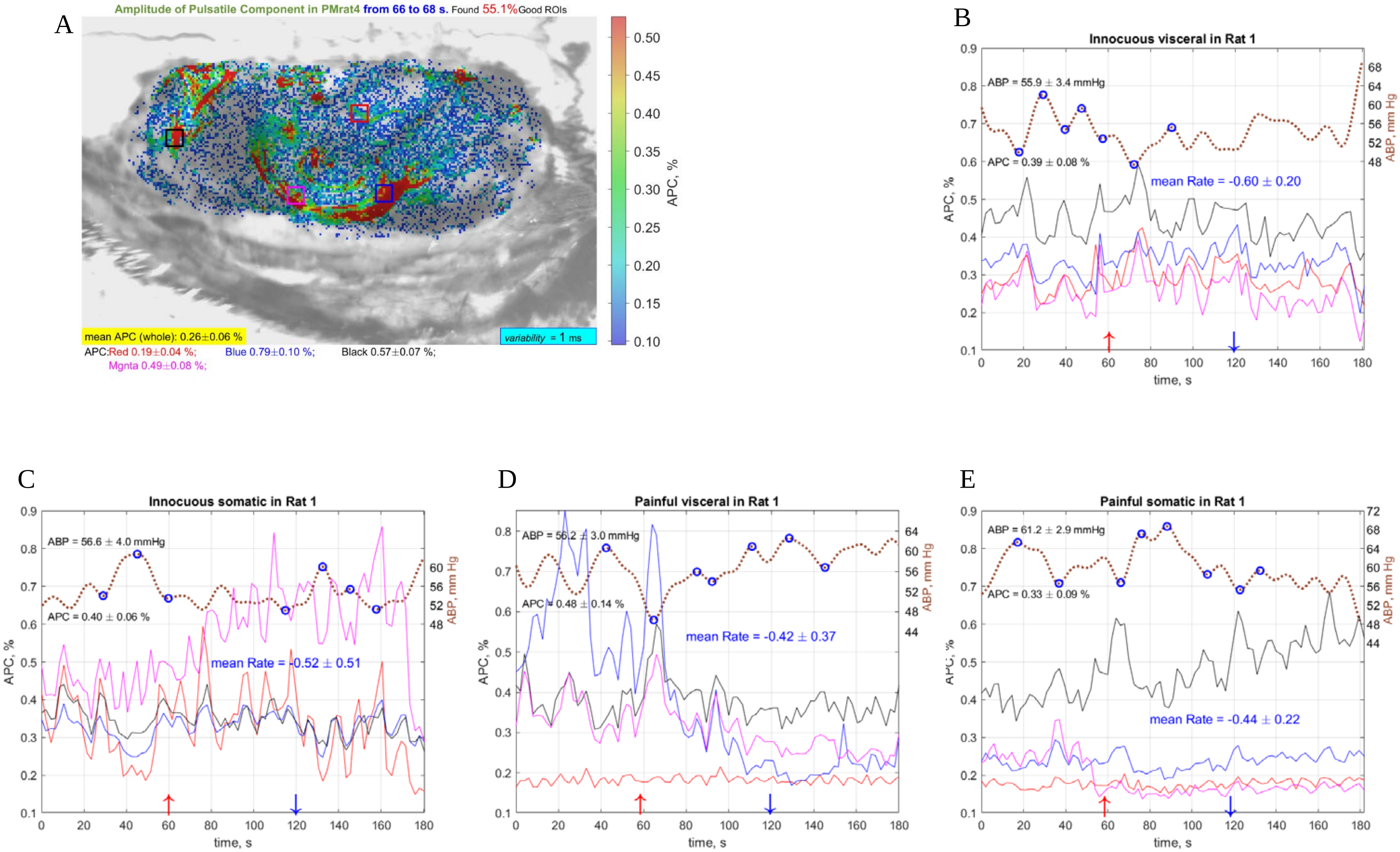

Figure S1. Rat No. 1 with dura mater (weight 348 g). Spatial distribution of APC over open brain cortex (A), and dynamics of ABP (brown dashed lines) and APC during functional stimulations: (B) innocuous visceral, (C) innocuous somatic, (D) painful visceral, and (E) painful somatic. Solid colored lines in graphs B-E show APC measured in ROIs, which positions are shown by squares of the same color in the panel (A).

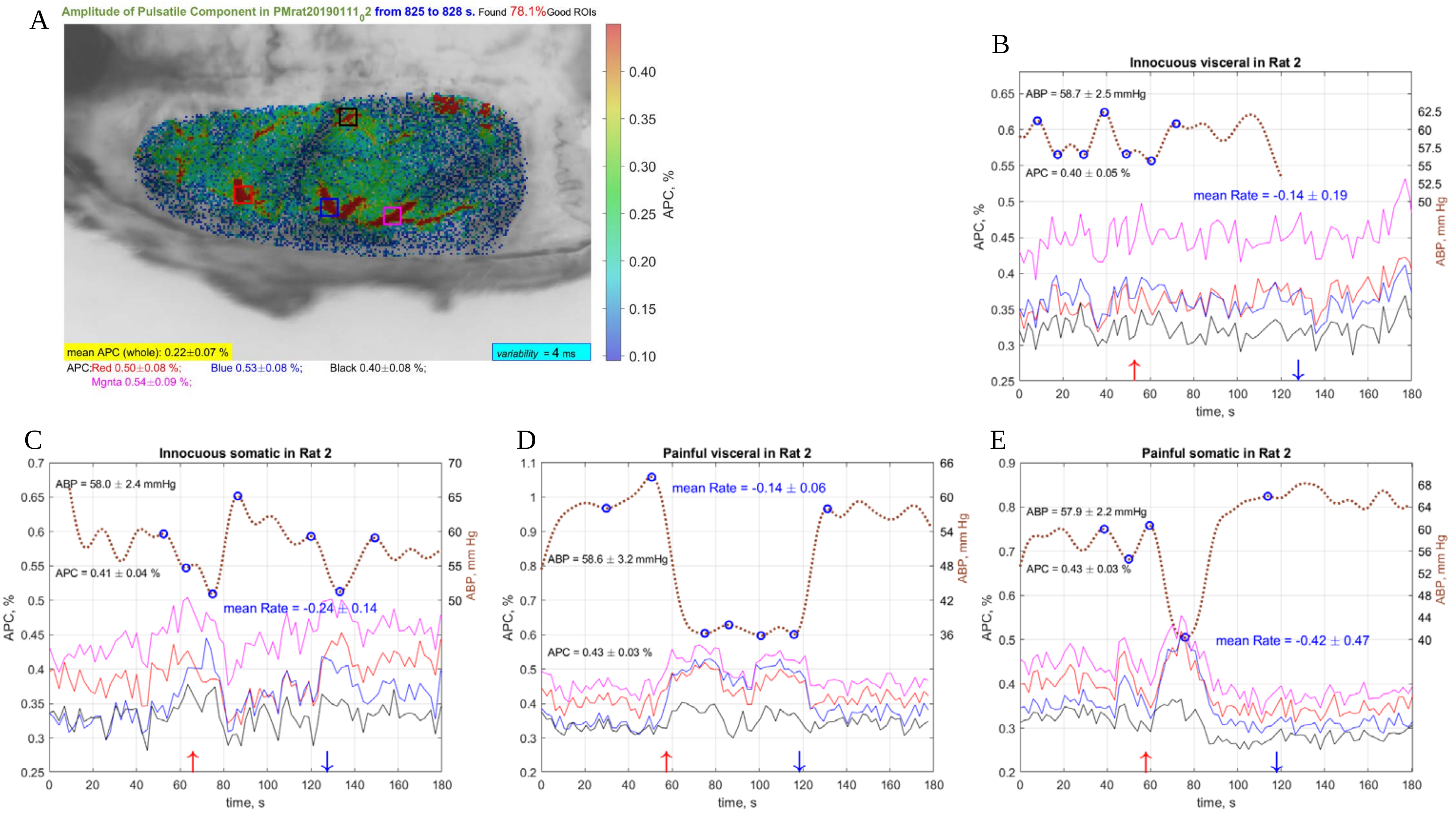

Figure S2. Rat No. 2 with dura mater (weight 322 g). Spatial distribution of APC over open brain cortex (A), and dynamics of ABP (brown dashed lines) and APC during functional stimulations: (B) innocuous visceral, (C) innocuous somatic, (D) painful visceral, and (E) painful somatic. Solid colored lines in graphs B-E show APC measured in ROIs, which positions are shown by squares of the same color in the panel (A).

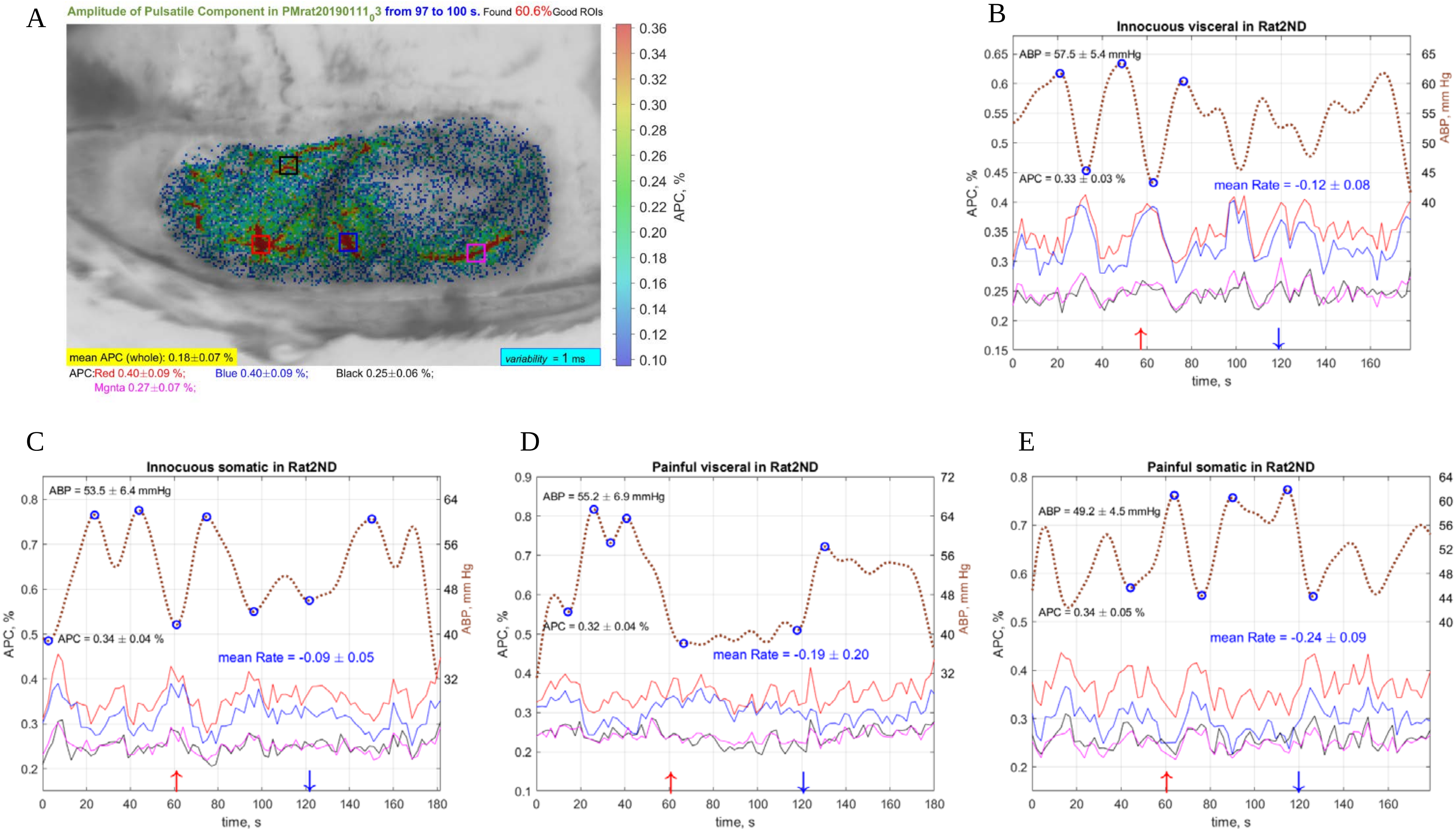

Figure S3. Rat No. 2 *without dura mater* (weight 322 g). Spatial distribution of APC over open brain cortex (A), and dynamics of ABP (brown dashed lines) and APC during functional stimulations: (B) innocuous visceral, (C) innocuous somatic, (D) painful visceral, and (E) painful somatic. Solid colored lines in graphs B-E show APC measured in ROIs, which positions are shown by squares of the same color in the panel (A).

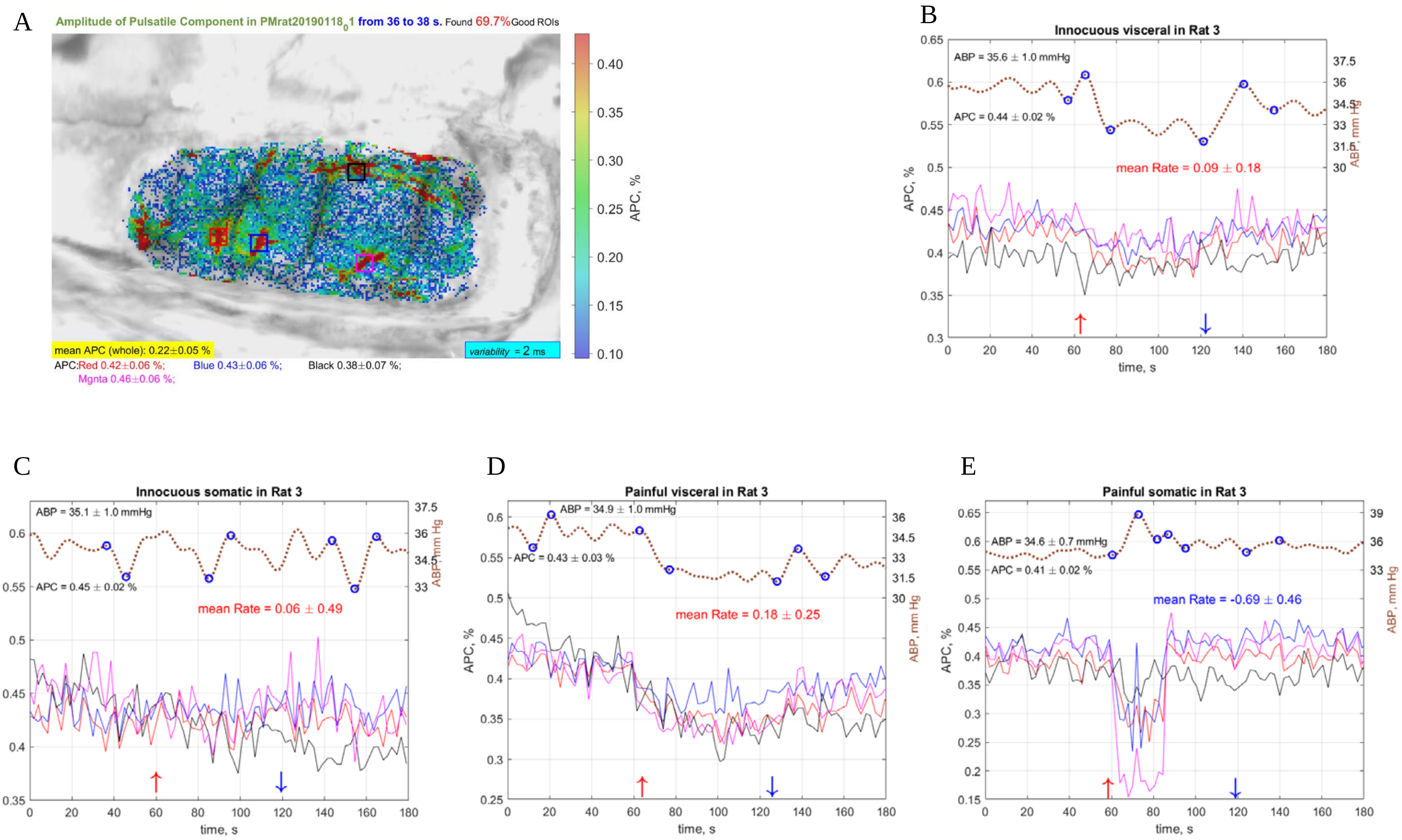

Figure S4. Rat No. 3 with dura mater (weight 290 g). Spatial distribution of APC over open brain cortex (A), and dynamics of ABP (brown dashed lines) and APC during functional stimulations: (B) innocuous visceral, (C) innocuous somatic, (D) painful visceral, and (E) painful somatic. Solid colored lines in graphs B-E show APC measured in ROIs, which positions are shown by squares of the same color in the panel (A).

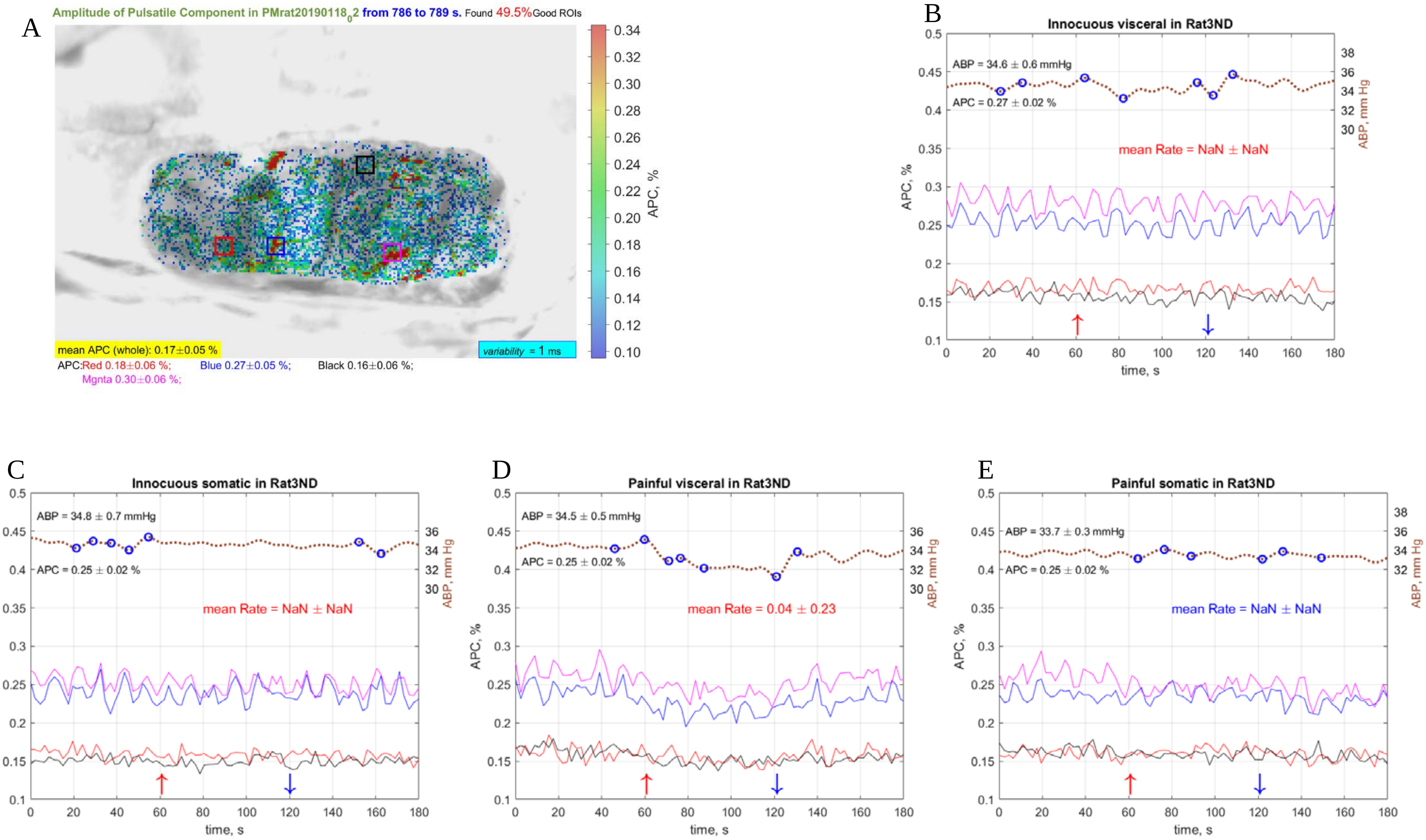

Figure S5. Rat No. 3 *without dura mater* (weight 290 g). Spatial distribution of APC over open brain cortex (A), and dynamics of ABP (brown dashed lines) and APC during functional stimulations: (B) innocuous visceral, (C) innocuous somatic, (D) painful visceral, and (E) painful somatic. Solid colored lines in graphs B-E show APC measured in ROIs, which positions are shown by squares of the same color in the panel (A).

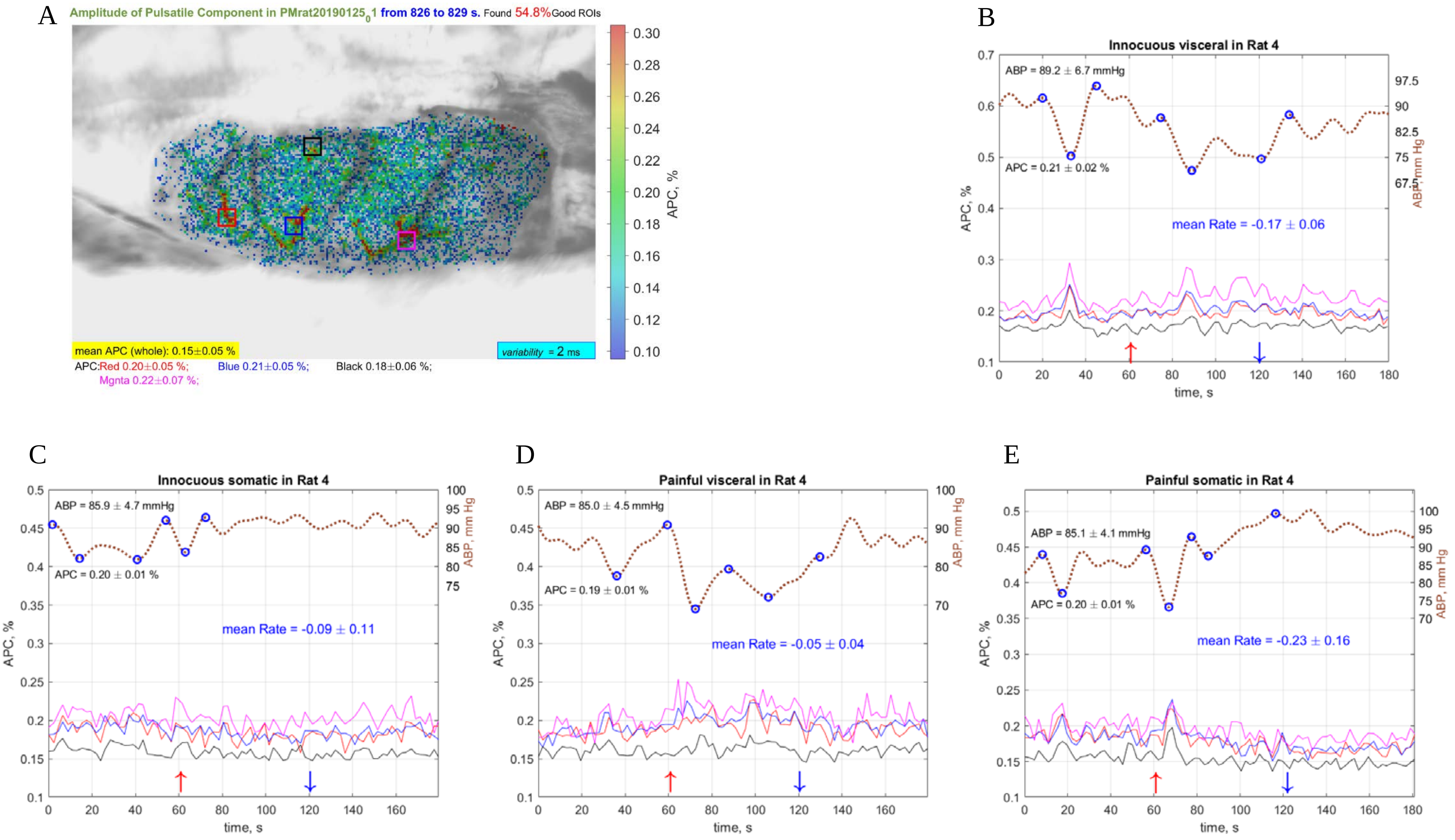

Figure S6. Rat No. 4 with dura mater (weight 320 g). Spatial distribution of APC over open brain cortex (A), and dynamics of ABP (brown dashed lines) and APC during functional stimulations: (B) innocuous visceral, (C) innocuous somatic, (D) painful visceral, and (E) painful somatic. Solid colored lines in graphs B-E show APC measured in ROIs, which positions are shown by squares of the same color in the panel (A).

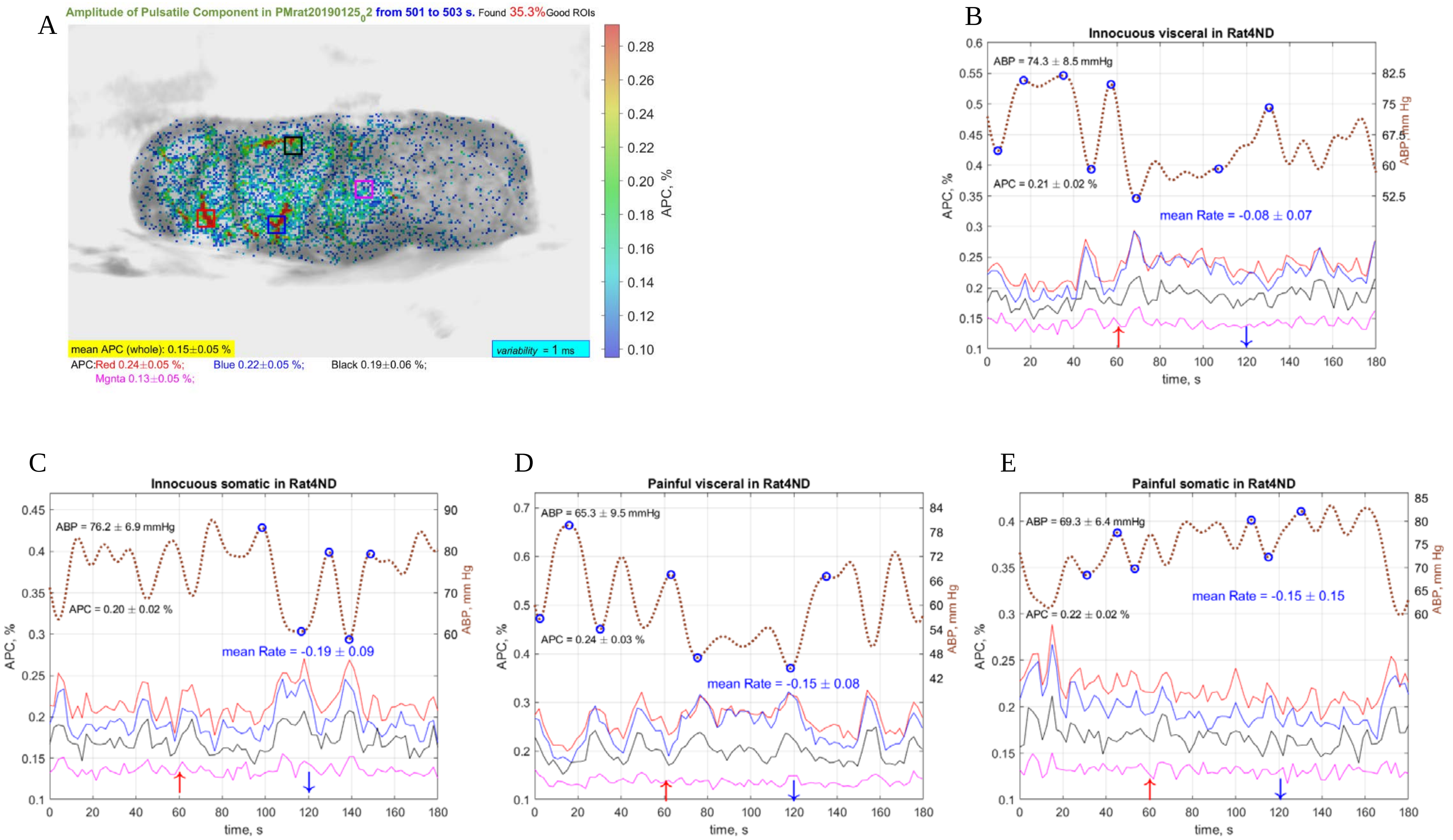

Figure S7. Rat No. 4 _without dura mater_ (weight 320 g). Spatial distribution of APC over open brain cortex (A), and dynamics of ABP (brown dashed lines) and APC during functional stimulations: (B) innocuous visceral, (C) innocuous somatic, (D) painful visceral, and (E) painful somatic. Solid colored lines in graphs B-E show APC measured in ROIs, which positions are shown by squares of the same color in the panel (A).

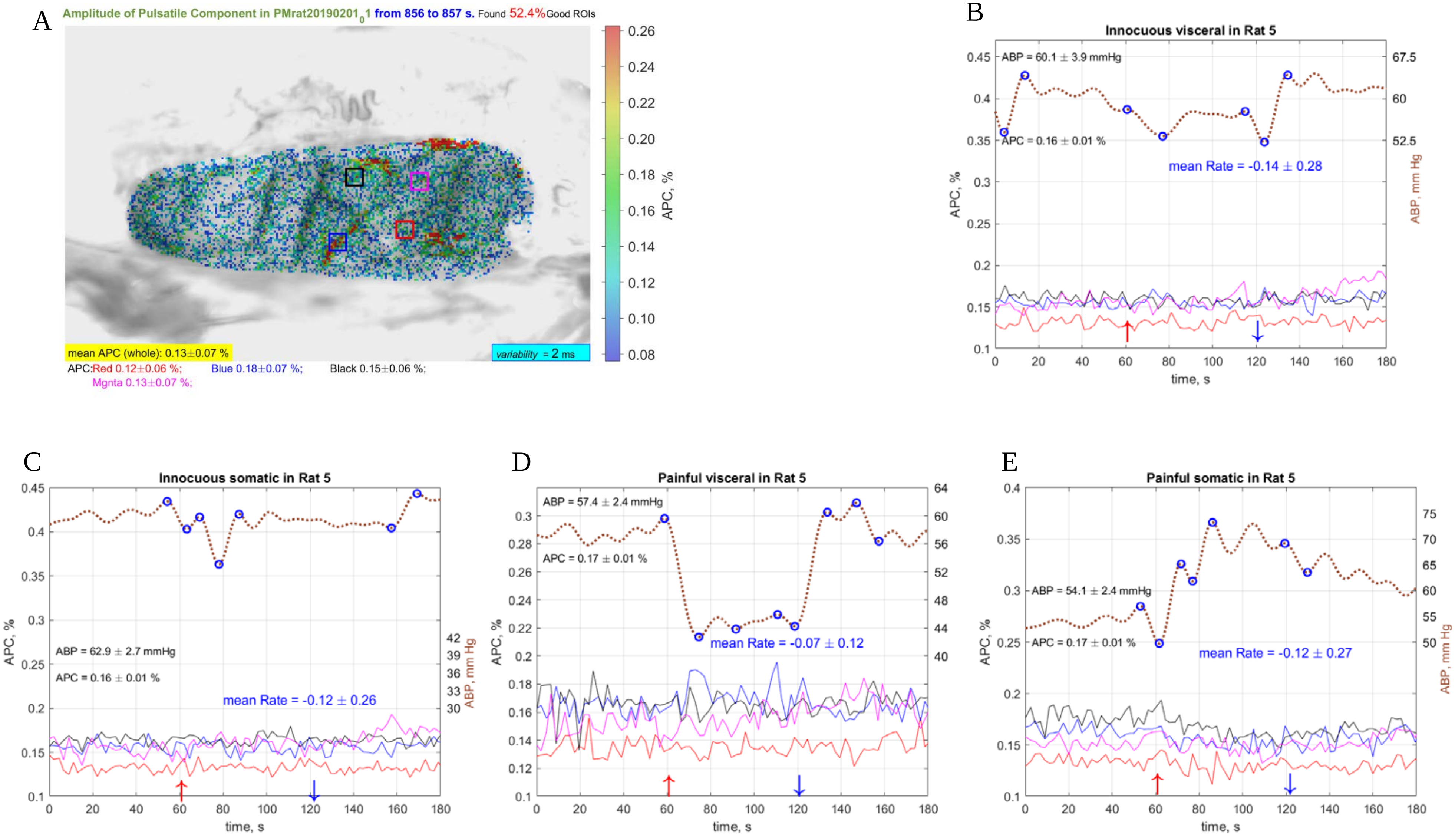

Figure S8. Rat No. 5 <u>with dura mater</u> (weight 346 g). Spatial distribution of APC over open brain cortex (A), and dynamics of ABP (brown dashed lines) and APC during functional stimulations: (B) innocuous visceral, (C) innocuous somatic, (D) painful visceral, and (E) painful somatic. Solid colored lines in graphs B-E show APC measured in ROIs, which positions are shown by squares of the same color in the panel (A).

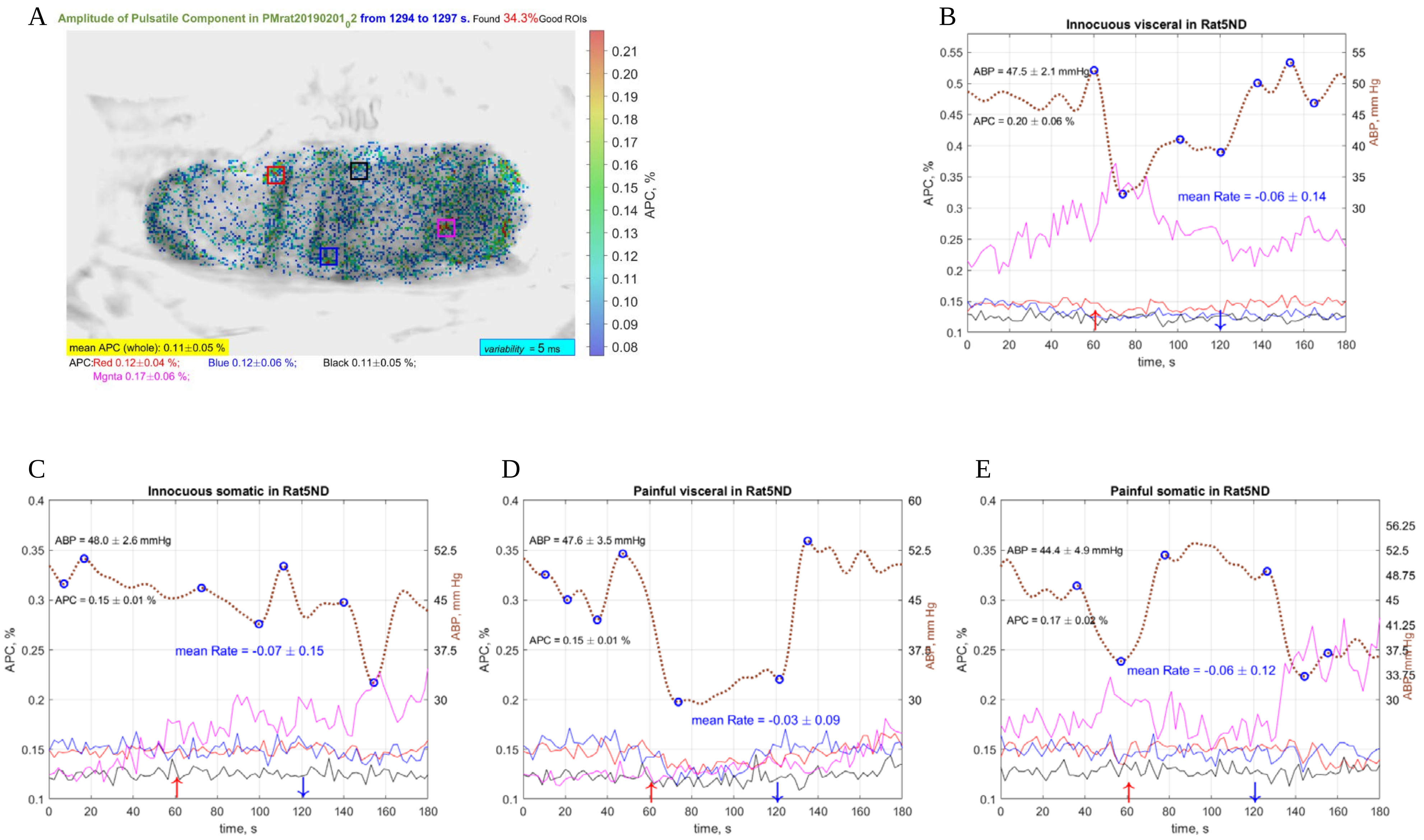

Figure S9. Rat No. 5 *without dura mater* (weight 346 g). Spatial distribution of APC over open brain cortex (A), and dynamics of ABP (brown dashed lines) and APC during functional stimulations: (B) innocuous visceral, (C) innocuous somatic, (D) painful visceral, and (E) painful somatic. Solid colored lines in graphs B-E show APC measured in ROIs, which positions are shown by squares of the same color in the panel (A).

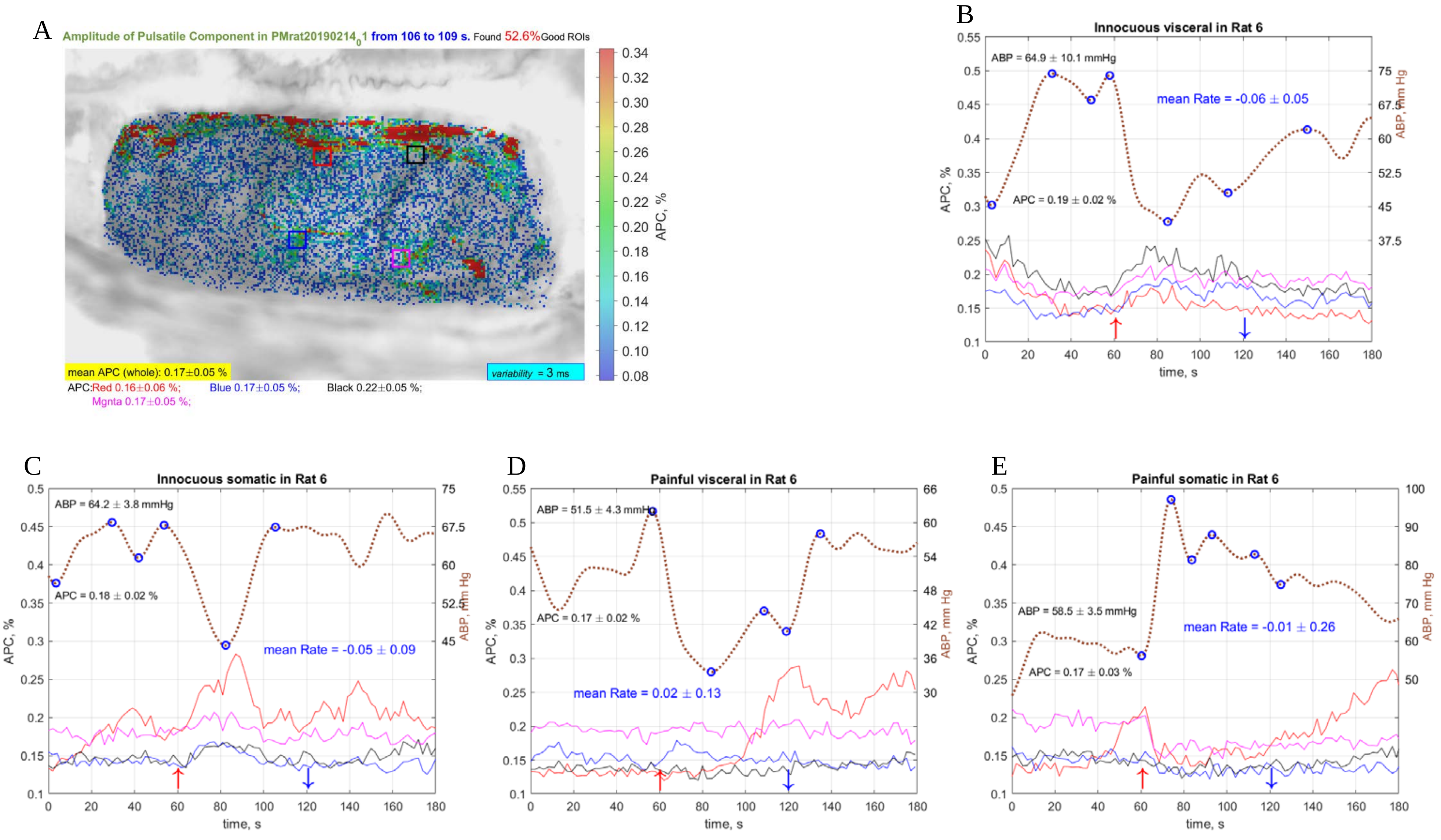

Figure S10. Rat No. 6 with dura mater (weight 399 g). Spatial distribution of APC over open brain cortex (A), and dynamics of ABP (brown dashed lines) and APC during functional stimulations: (B) innocuous visceral, (C) innocuous somatic, (D) painful visceral, and (E) painful somatic. Solid colored lines in graphs B-E show APC measured in ROIs, which positions are shown by squares of the same color in the panel (A).

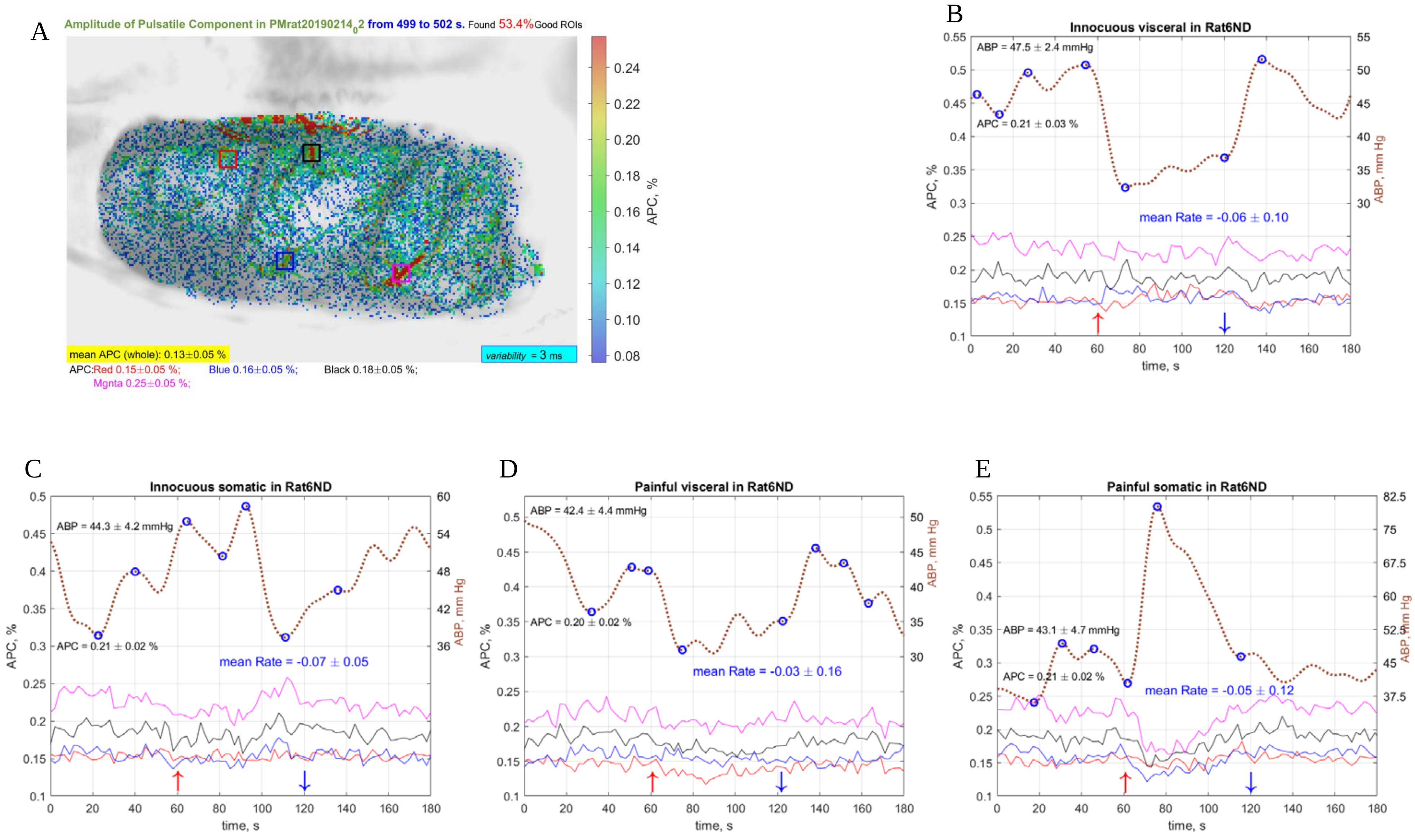

Figure S11. Rat No. 6 *without dura mater* (weight 399 g). Spatial distribution of APC over open brain cortex (A), and dynamics of ABP (brown dashed lines) and APC during functional stimulations: (B) innocuous visceral, (C) innocuous somatic, (D) painful visceral, and (E) painful somatic. Solid colored lines in graphs B-E show APC measured in ROIs, which positions are shown by squares of the same color in the panel (A).

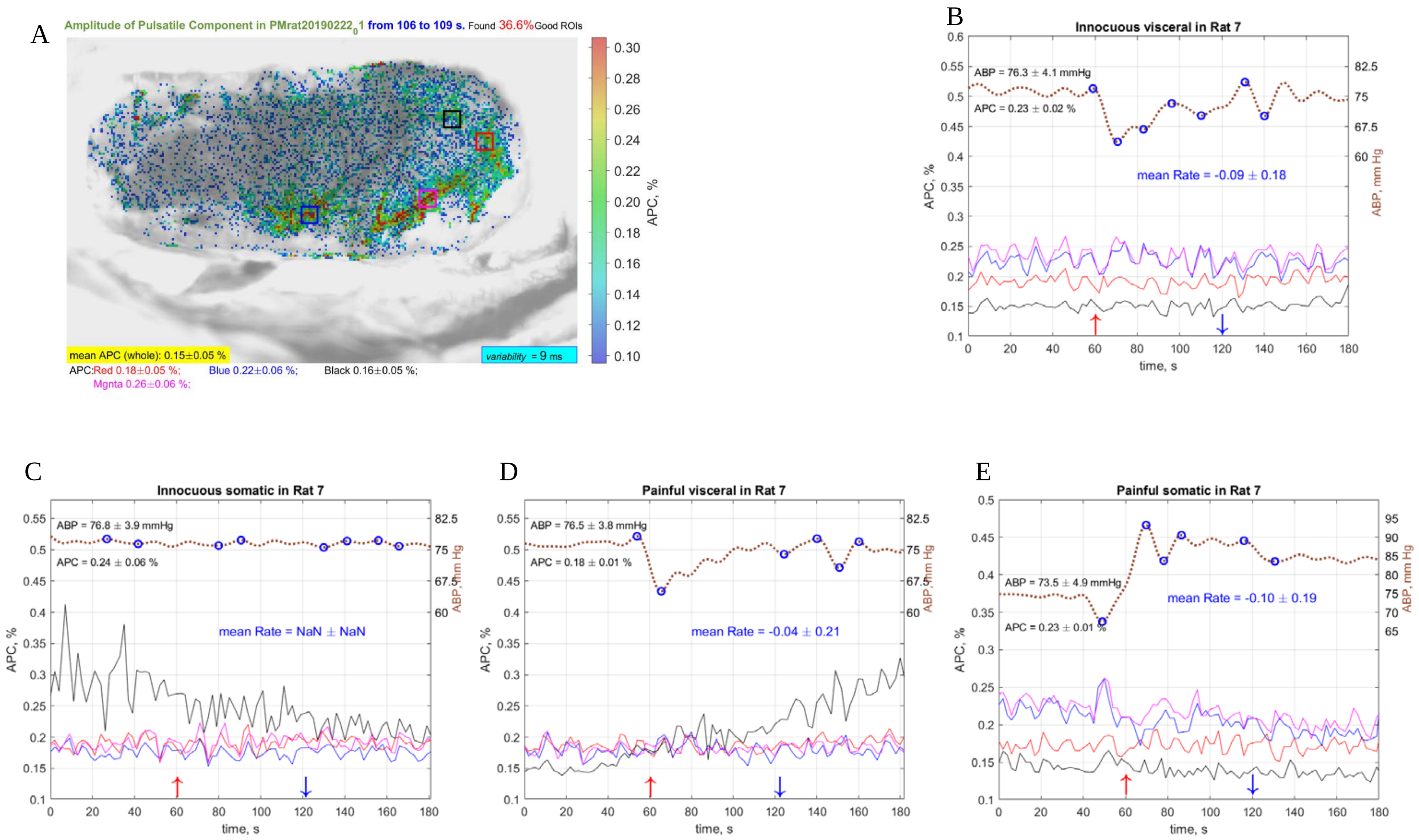

Figure S12. Rat No. 7 with dura mater (weight 438 g). Spatial distribution of APC over open brain cortex (A), and dynamics of ABP (brown dashed lines) and APC during functional stimulations: (B) innocuous visceral, (C) innocuous somatic, (D) painful visceral, and (E) painful somatic. Solid colored lines in graphs B-E show APC measured in ROIs, which positions are shown by squares of the same color in the panel (A).

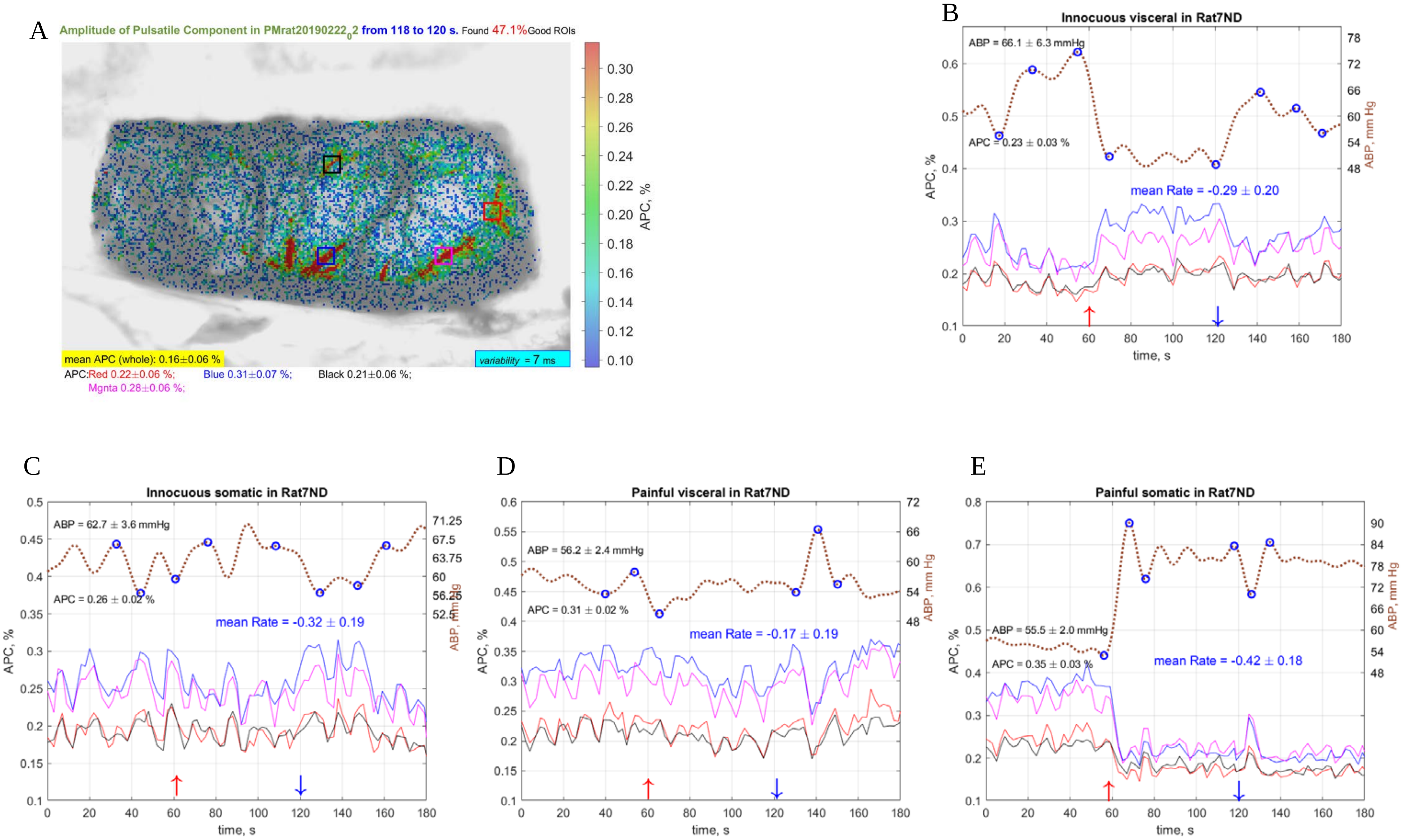

Figure S13. Rat No. 7 *without dura mater* (weight 438 g). Spatial distribution of APC over open brain cortex (A), and dynamics of ABP (brown dashed lines) and APC during functional stimulations: (B) innocuous visceral, (C) innocuous somatic, (D) painful visceral, and (E) painful somatic. Solid colored lines in graphs B-E show APC measured in ROIs, which positions are shown by squares of the same color in the panel (A).

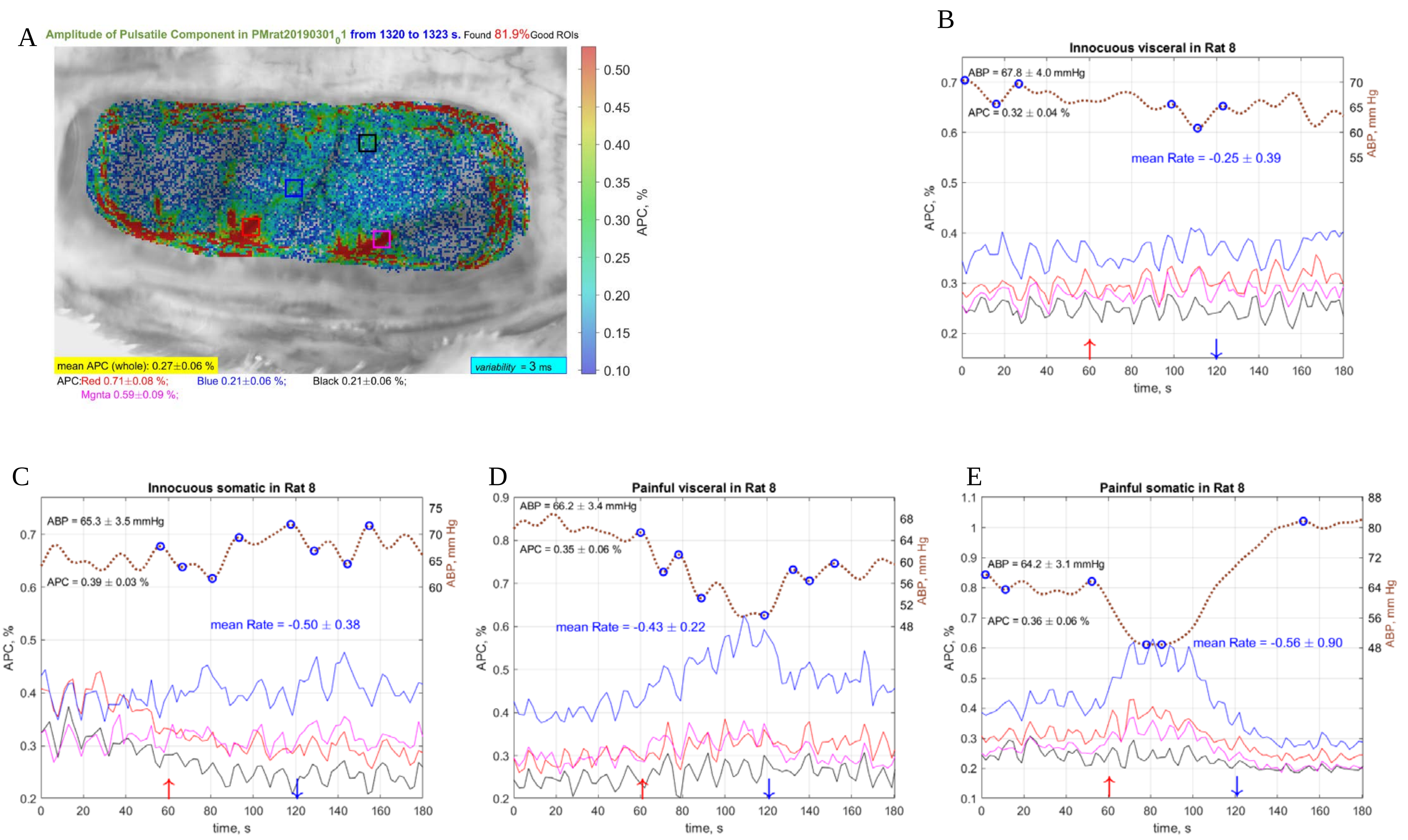

Figure S14. Rat No. 8 with dura mater (weight 290 g). Spatial distribution of APC over open brain cortex (A), and dynamics of ABP (brown dashed lines) and APC during functional stimulations: (B) innocuous visceral, (C) innocuous somatic, (D) painful visceral, and (E) painful somatic. Solid colored lines in graphs B-E show APC measured in ROIs, which positions are shown by squares of the same color in the panel (A).

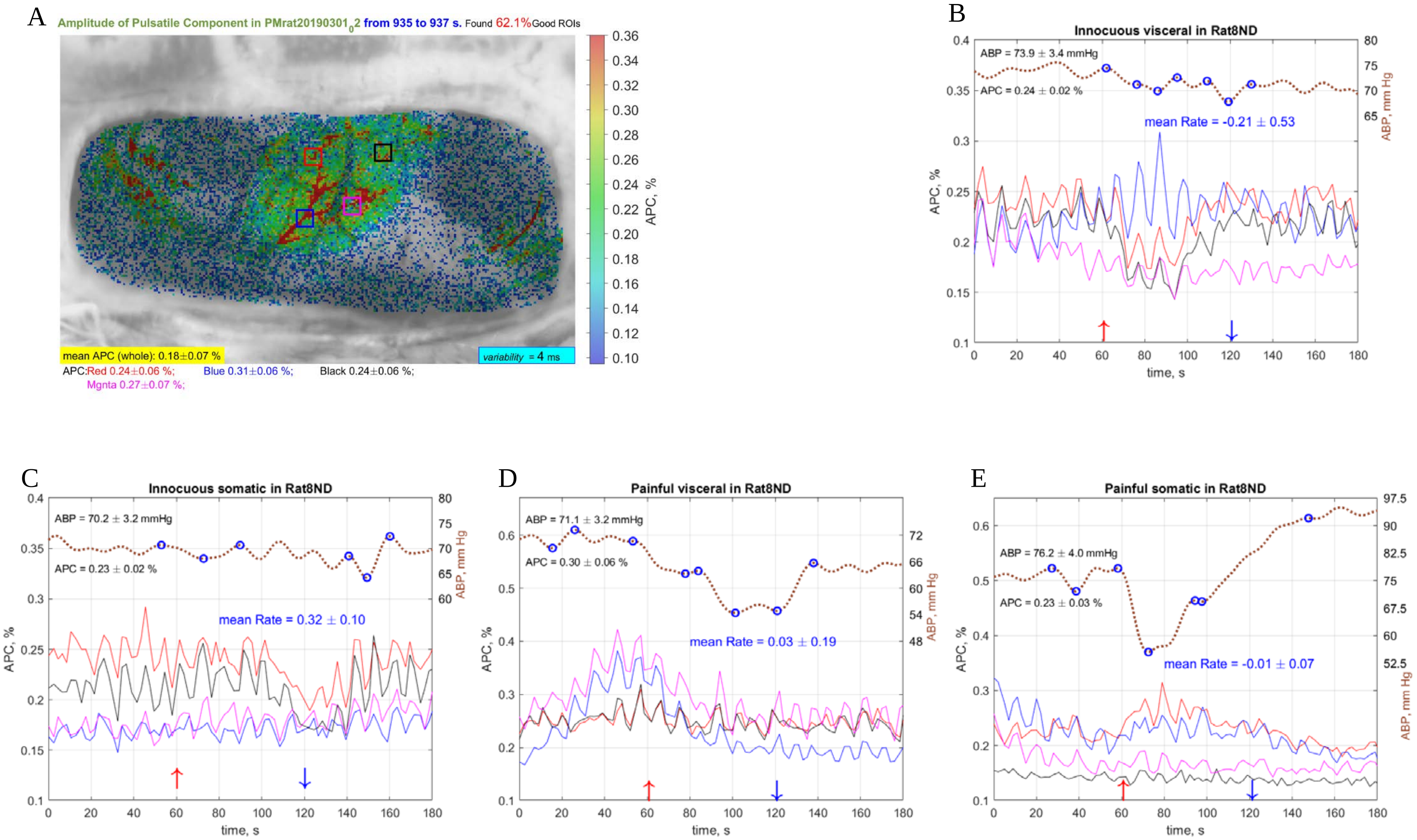

Figure S15. Rat No. 8 *without dura mater* (weight 290 g). Spatial distribution of APC over open brain cortex (A), and dynamics of ABP (brown dashed lines) and APC during functional stimulations: (B) innocuous visceral, (C) innocuous somatic, (D) painful visceral, and (E) painful somatic. Solid colored lines in graphs B-E show APC measured in ROIs, which positions are shown by squares of the same color in the panel (A).

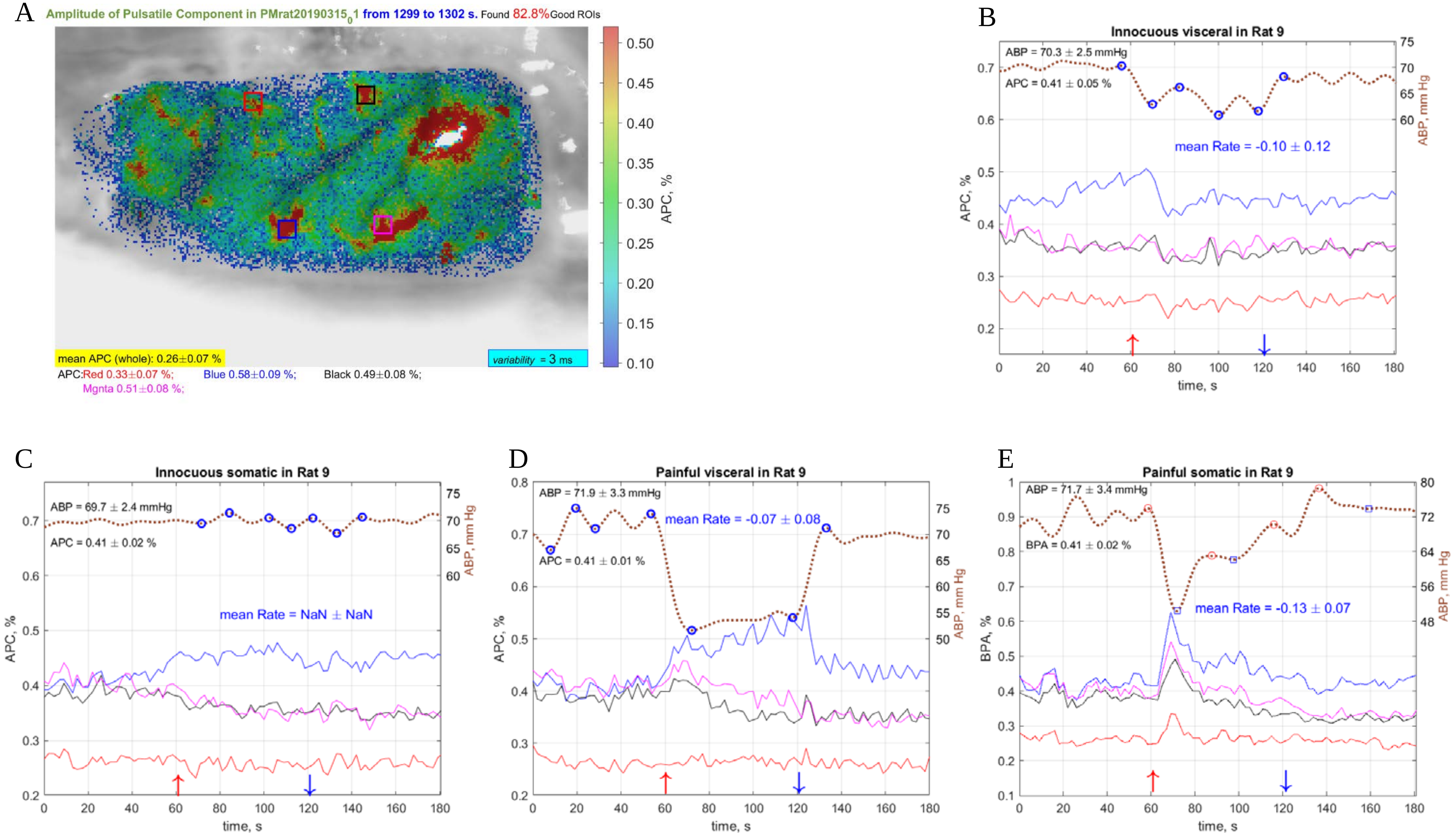

Figure S16. Rat No. 9 with dura mater (weight 308 g). Spatial distribution of APC over open brain cortex (A), and dynamics of ABP (brown dashed lines) and APC during functional stimulations: (B) innocuous visceral, (C) innocuous somatic, (D) painful visceral, and (E) painful somatic. Solid colored lines in graphs B-E show APC measured in ROIs, which positions are shown by squares of the same color in the panel (A).

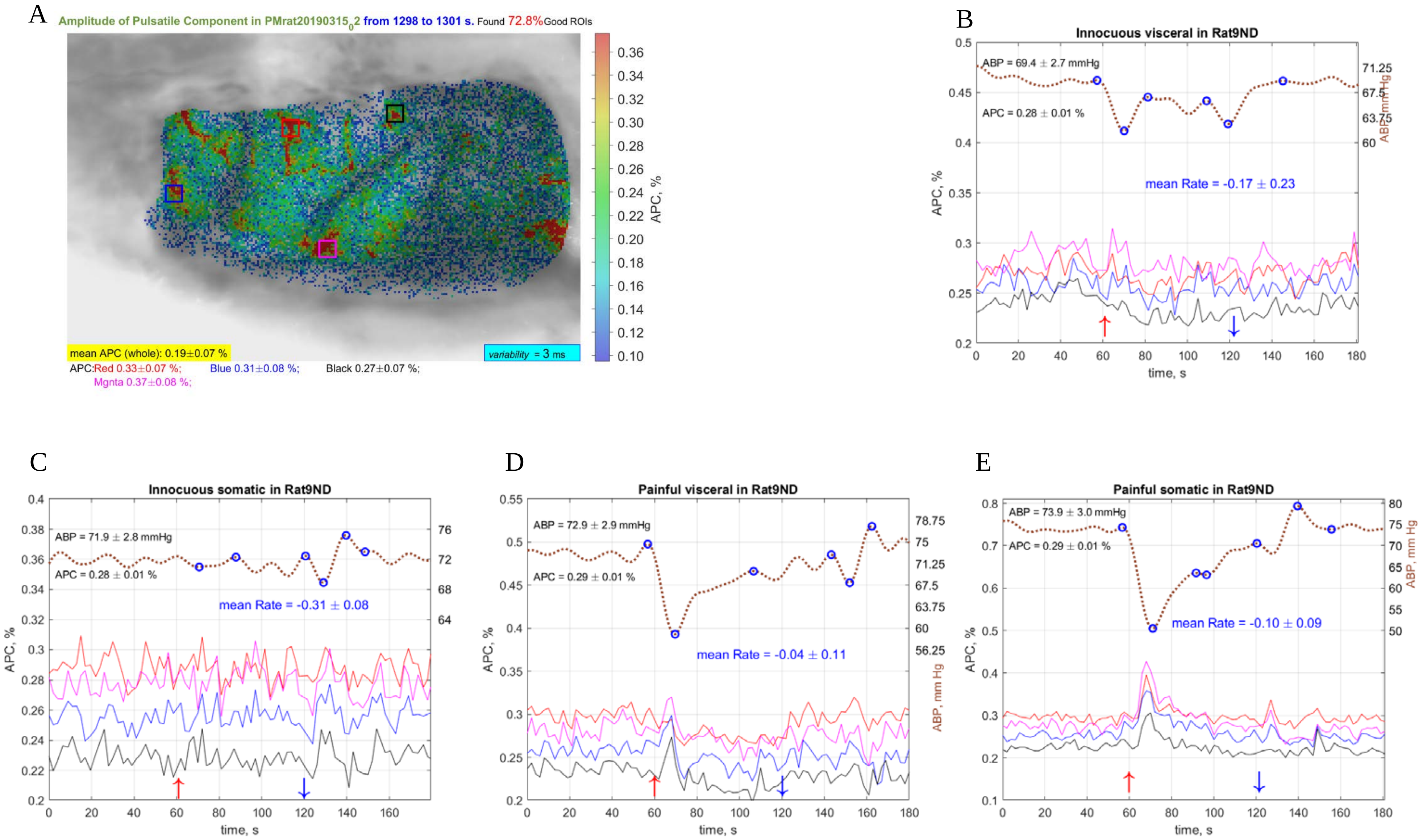

Figure S17. Rat No. 9 *without dura mater* (weight 308 g). Spatial distribution of APC over open brain cortex (A), and dynamics of ABP (brown dashed lines) and APC during functional stimulations: (B) innocuous visceral, (C) innocuous somatic, (D) painful visceral, and (E) painful somatic. Solid colored lines in graphs B-E show APC measured in ROIs, which positions are shown by squares of the same color in the panel (A).

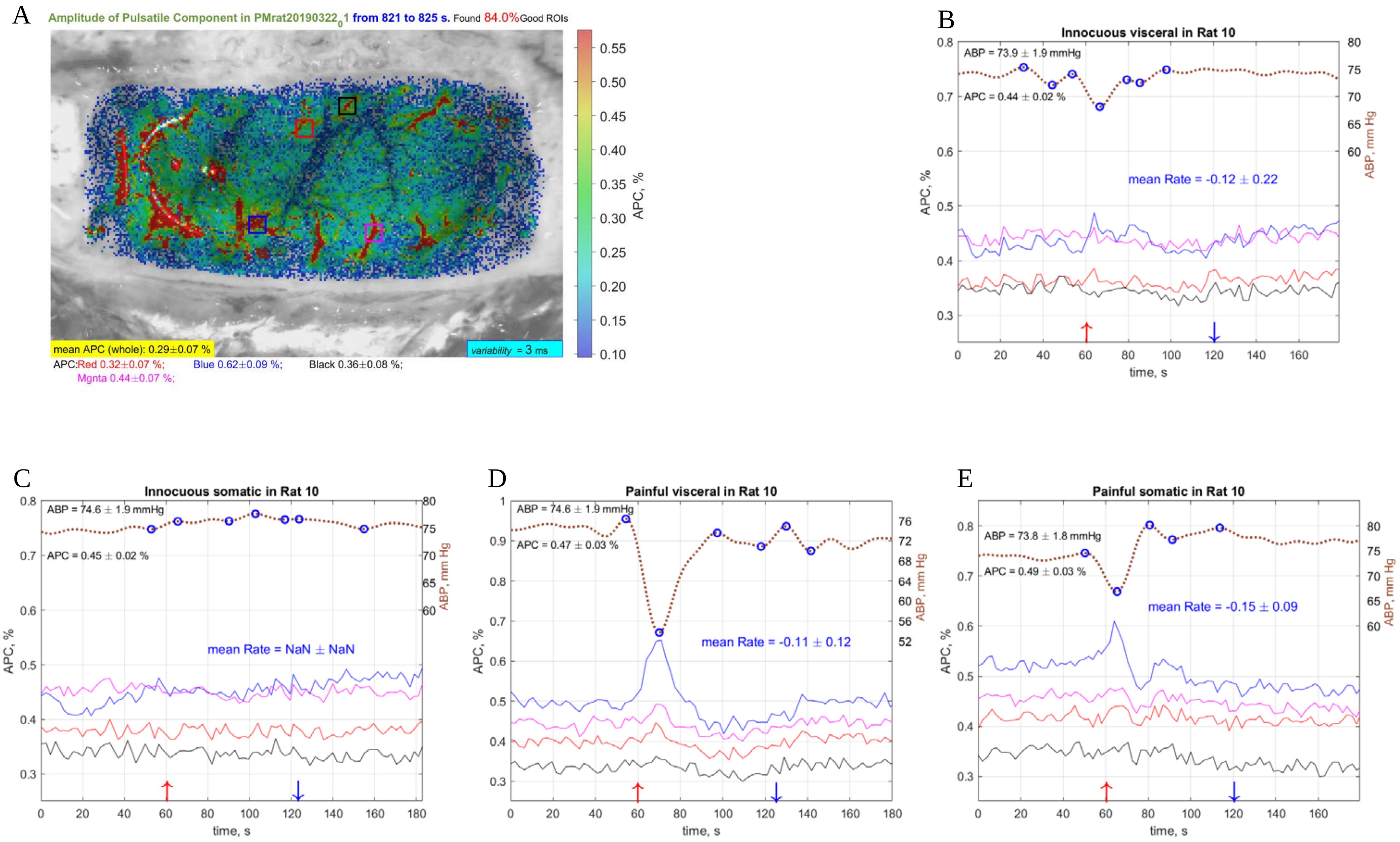

Figure S18. Rat No. 10 with dura mater (weight 280 g). Spatial distribution of APC over open brain cortex (A), and dynamics of ABP (brown dashed lines) and APC during functional stimulations: (B) innocuous visceral, (C) innocuous somatic, (D) painful visceral, and (E) painful somatic. Solid colored lines in graphs B-E show APC measured in ROIs, which positions are shown by squares of the same color in the panel (A).

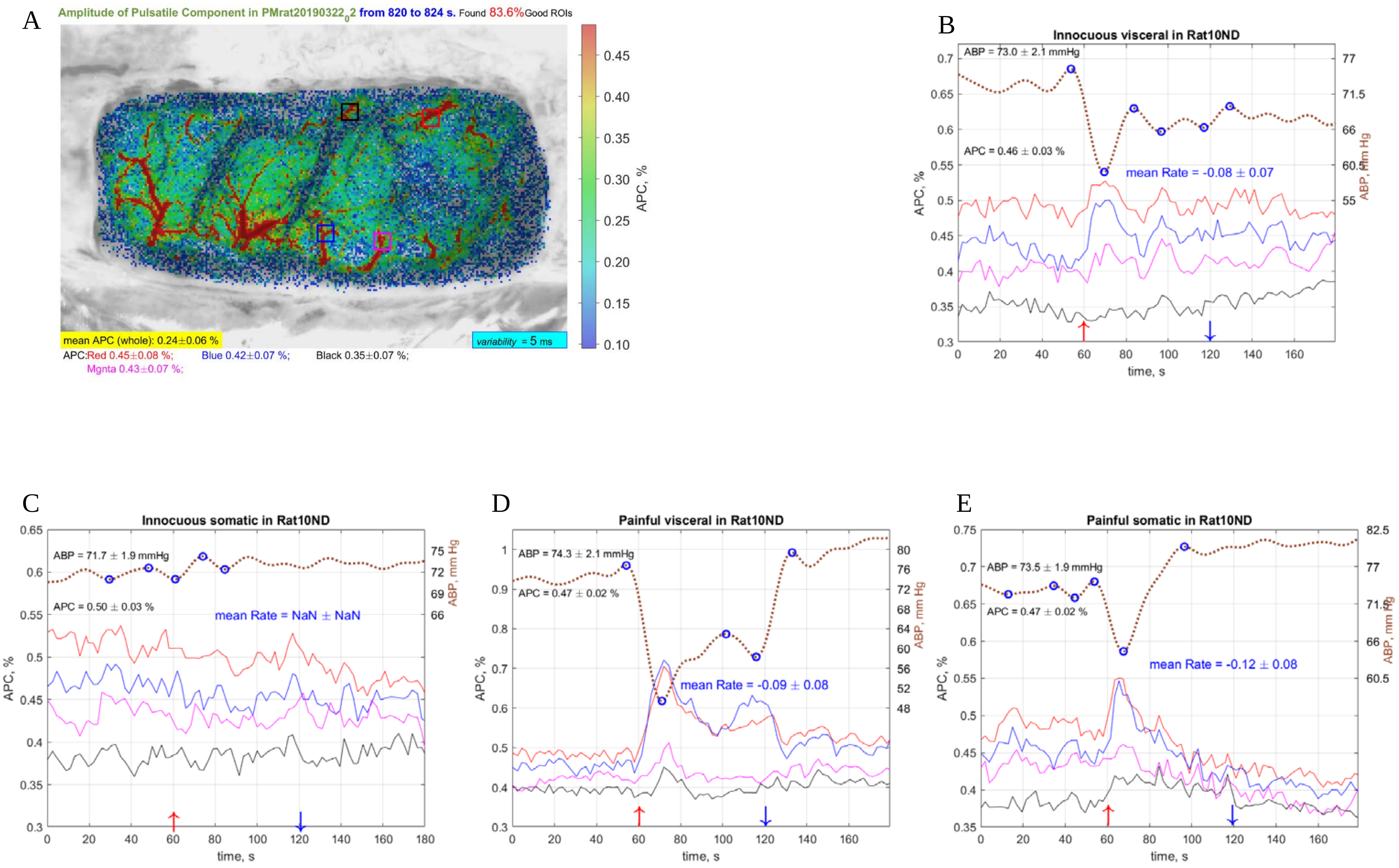

Figure S19. Rat No. 10 *without dura mater* (weight 280 g). Spatial distribution of APC over open brain cortex (A), and dynamics of ABP (brown dashed lines) and APC during functional stimulations: (B) innocuous visceral, (C) innocuous somatic, (D) painful visceral, and (E) painful somatic. Solid colored lines in graphs B-E show APC measured in ROIs, which positions are shown by squares of the same color in the panel (A).

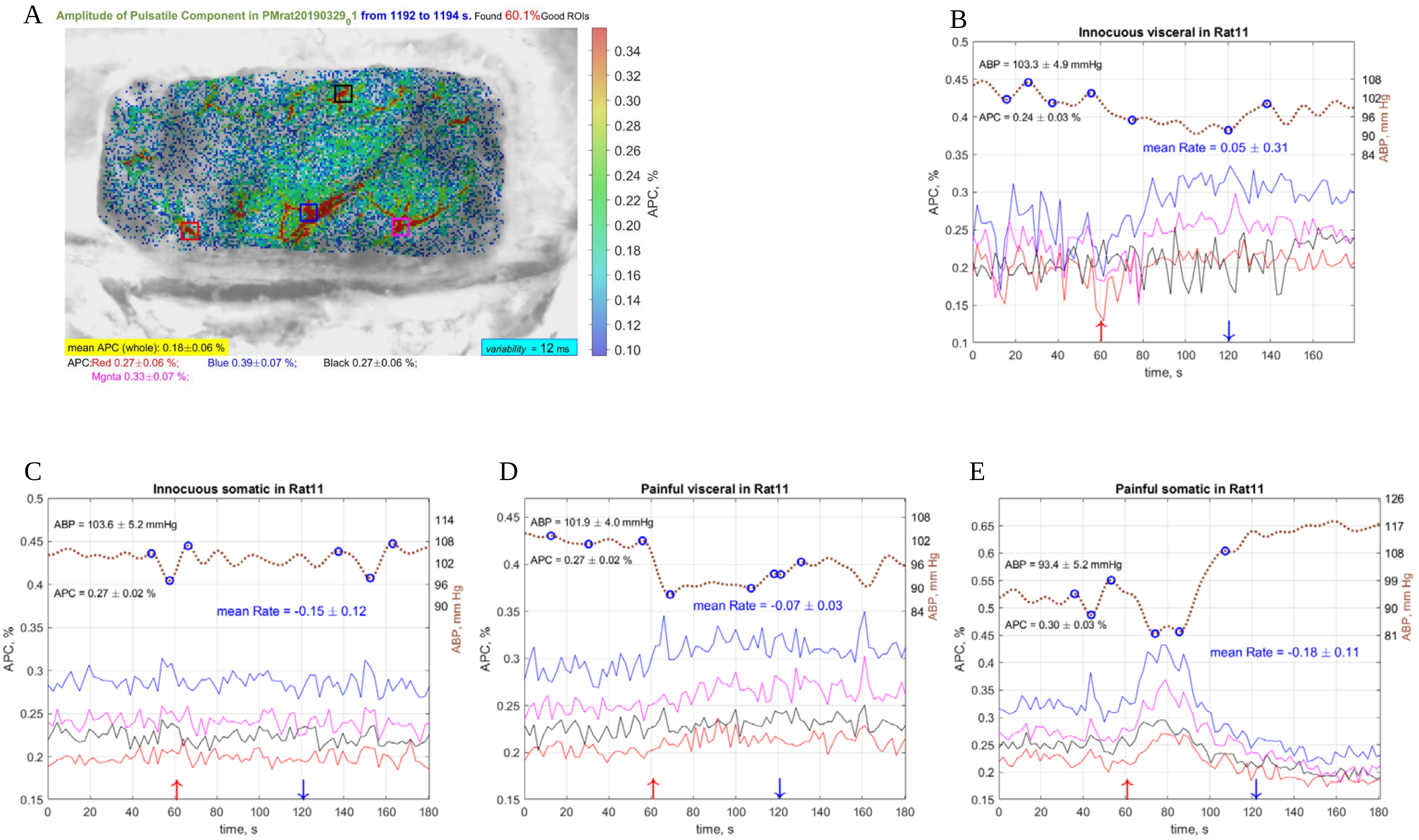

Figure S20. Rat No. 11 with dura mater (weight 260 g). Spatial distribution of APC over open brain cortex (A), and dynamics of ABP (brown dashed lines) and APC during functional stimulations: (B) innocuous visceral, (C) innocuous somatic, (D) painful visceral, and (E) painful somatic. Solid colored lines in graphs B-E show APC measured in ROIs, which positions are shown by squares of the same color in the panel (A).

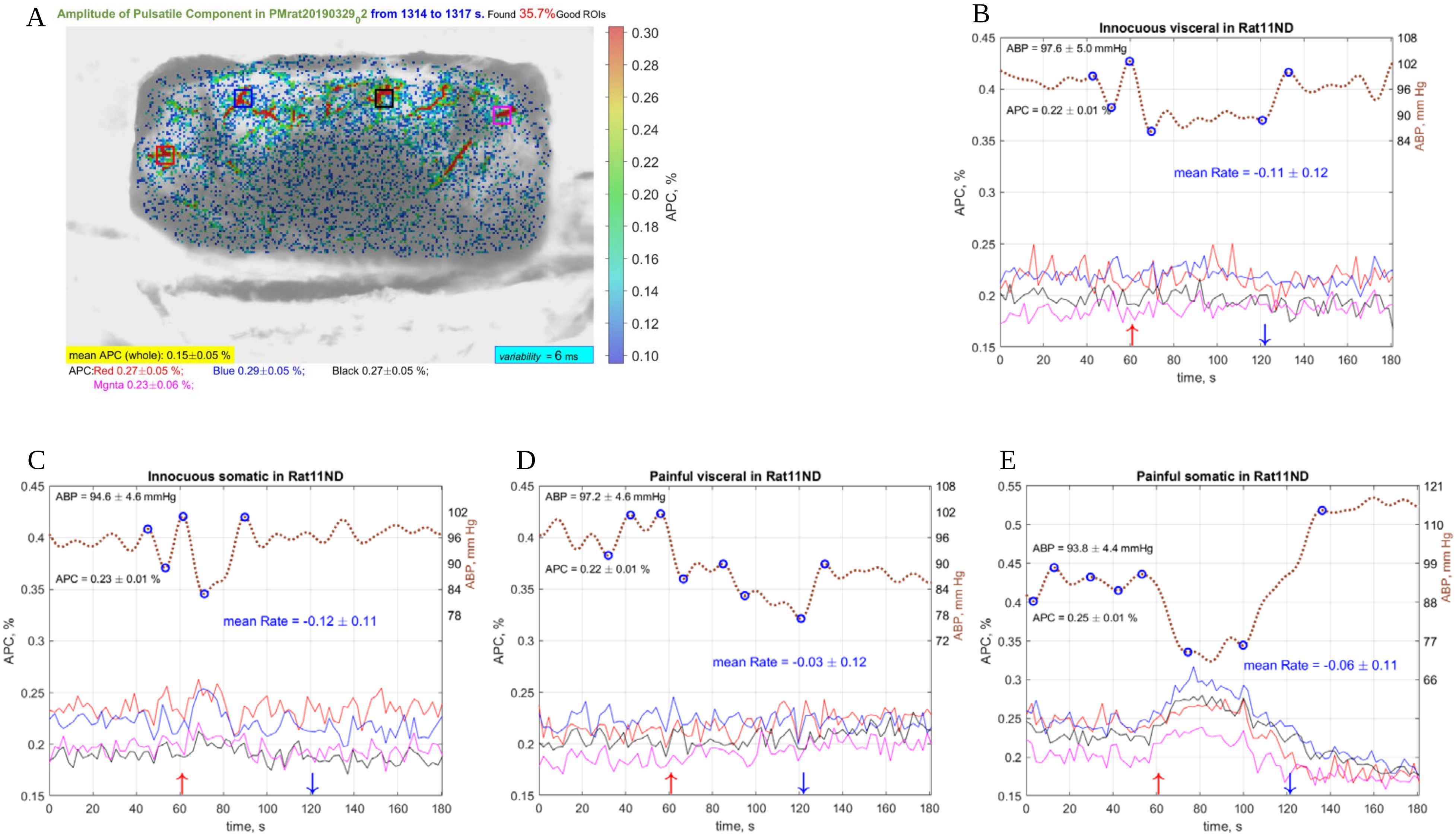

Figure S21. Rat No. 11 *without dura mater* (weight 260 g). Spatial distribution of APC over open brain cortex (A), and dynamics of ABP (brown dashed lines) and APC during functional stimulations: (B) innocuous visceral, (C) innocuous somatic, (D) painful visceral, and (E) painful somatic. Solid colored lines in graphs B-E show APC measured in ROIs, which positions are shown by squares of the same color in the panel (A).
